# PARP Inhibitors plus Anlotinib as Bridging Therapy for Armored CAR‐T Cells in Ovarian Cancer Enhances Infiltration and Antitumor Efficacy

**DOI:** 10.1002/advs.76994

**Published:** 2026-08-10

**Authors:** Huayi Li, Minghua Xiang, Qiuyang Xu, Jiahao Liu, Xiaofei Jiao, Wei Mu, Xiaoying Lv, Kangjia Tao, Yu Xu, Dongchen Zhou, Wenjian Gong, Danmei Yan, Yixin Hu, Sen Xu, Bingbing Zhao, Jundong Li, Yongkang Gai, Guang Hu, Li Zhu, Qinglei Gao, Yong Fang

**Affiliations:** ^1^ Department of Obstetrics and Gynecology National Clinical Research Center for Obstetrics and Gynecology Tongji Hospital Tongji Medical College Huazhong University of Science and Technology Wuhan China; ^2^ Department of Gynecologic Oncology Sun Yat‐sen University Cancer Center Guangzhou China; ^3^ Key Laboratory of Cancer Invasion and Metastasis (Ministry of Education), Hubei Key Laboratory of Tumor Invasion and Metastasis, Tongji Hospital, Tongji Medical College Huazhong University of Science and Technology Wuhan China; ^4^ State Key Laboratory of Oncology in South China Collaborative Innovation Center for Cancer Medicine Guangzhou China; ^5^ Department of Hematology Tongji Hospital Tongji Medical College Huazhong University of Science and Technology Wuhan China; ^6^ Department of Nuclear Medicine Union Hospital Tongji Medical College Huazhong University of Science and Technology Wuhan China; ^7^ Department of Gynecology and Obstetrics Union Hospital Tongji Medical College Huazhong University of Science and Technology Wuhan China; ^8^ Department of Gynecology and Obstetrics The First Affiliated Hospital (Southwest Hospital) of Army Medical University Chongqing China; ^9^ Department of Pharmaceutics School of Pharmacy Tongji Medical College Huazhong University of Science and Technology Wuhan China; ^10^ Department of Gynecologic Oncology Guangxi University Cancer Hospital Nanning China; ^11^ Nanjing IASO Biotherapeutics Ltd. Nanjing China

**Keywords:** anlotinib, bridging therapy, CAR‐T cell therapy, ovarian cancer, PARP inhibitor

## Abstract

Chimeric antigen receptor (CAR)‐T cell therapy is largely ineffective in most solid tumors, partially because of inadequate intratumoral trafficking. Bridging therapy, administered between leukapheresis and CAR‐T cell infusion, offers a distinct opportunity to control the disease and precondition the tumor microenvironment without directly exposing CAR‐T cells to concomitant drugs. Here, a poly (ADP‐ribose) polymerase (PARP) inhibitor combined with anlotinib is evaluated as bridging therapy for ovarian cancer. In immunocompetent mice bearing ovarian tumors, this regimen induces durable intratumoral T cell accumulation associated with activation of the cGAS‐STING pathway, vascular normalization, and reduced fibrillar collagen deposition. Furthermore, TGFβ‐resistant, dual‐target mesothelin (MSLN)/CD19 CAR‐T cells are engineered using a bait‐and‐switch strategy. Niraparib plus anlotinib administered as bridging therapy before CAR‐T cell infusion enhances CAR‐T cell infiltration and antitumor activity across multiple preclinical ovarian cancer models. These findings inform an ongoing phase I clinical trial (NCT05141253) evaluating the safety of these engineered CAR‐T cells following bridging therapy in patients with refractory MSLN‐positive solid tumors. Collectively, our findings support a clinically actionable bridging therapy with translational potential for potentiating CAR‐T cell therapy in ovarian cancer and highlight bridging therapy as a translational approach to improve CAR‐T cell access and efficacy in solid tumors.

## Graphical abstract

This study shows that a PARP inhibitor combined with anlotinib, administered as bridging therapy, preconditions the tumor microenvironment to enhance the infiltration and antitumor activity of subsequently infused CAR‐T cells across preclinical ovarian cancer models, supporting bridging therapy as a promising strategy to address limited CAR‐T cell trafficking and improve therapeutic efficacy in solid tumors.

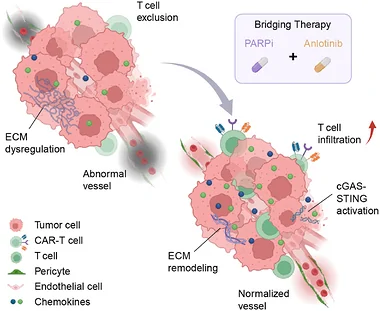

## 1 Introduction

Chimeric antigen receptor (CAR)‐T cell therapy reprograms patients’ T cells to selectively recognize and eradicate tumor cells, functioning as a living drug capable of sustained immune surveillance [1]. This approach has revolutionized the treatment of hematologic malignancies, producing durable remission and prolonged disease control in patients once considered untreatable [2, 3, 4]. However, translating this success to solid tumors remains a major challenge in oncology [5]. A major obstacle is the inadequate infiltration of CAR‐T cells into the tumor microenvironment (TME), largely attributable to aberrant tumor vasculature, a dense extracellular matrix (ECM), and chemokine mismatches [6, 7]. Overcoming these barriers is therefore essential to fully unleash the therapeutic potential of CAR‐T cells in solid tumors.

Combination strategies leveraging the immunomodulatory properties of clinically approved agents have emerged as promising approaches to enhance CAR‐T cell efficacy in solid tumors [8]. Poly (ADP‐ribose) polymerase (PARP) inhibitors, which are widely used in homologous recombination deficiency (HRD)‐positive ovarian cancer [9, 10], can enhance T cell infiltration by inducing immunogenic cell death and activating the cGAS‐STING pathway [11, 12], thereby providing a strong rationale for their integration with CAR‐T cells. However, our previous analyses of paired tumor samples obtained before and after PARP inhibitor treatment revealed direct toxicity toward T cells, characterized by DNA damage accumulation and impaired proliferative capacity [13, 14]. Consistently, co‐administration of a PARP inhibitor did not improve survival in tumor‐bearing mice treated with CAR‐T cells [14]. These observations highlight the need for approaches that preserve the immunostimulatory advantages of PARP inhibition while minimizing collateral toxicity to CAR‐T cells.

Bridging therapy, administered between leukapheresis and CAR‐T cell infusion, offers a distinct therapeutic window. This allows for restraining disease progression, avoiding temporal overlap with infused CAR‐T cells, and preconditioning the TME to favor subsequent CAR‐T cell activity [15]. Increasing evidence indicates that the immune landscape at the time of CAR‐T cell infusion is a critical determinant of therapeutic response [16, 17, 18, 19, 20]. However, it remains unclear whether appropriately designed bridging therapy can enhance CAR‐T cell efficacy in solid tumors. Ovarian cancer, the leading cause of death among gynecologic malignancies [21], is highly prone to chemoresistance, underscoring the urgent need for innovative treatment strategies [22]. The ANNIE trial reported encouraging clinical activity of niraparib plus anlotinib in patients with platinum‐resistant recurrent ovarian cancer [23]. Niraparib has an elimination half‐life of approximately 36 h following multiple 300‐mg daily doses, facilitating timely clearance before CAR‐T cell infusion [24]. Anlotinib is an orally administered multitarget receptor tyrosine kinase inhibitor with activity against vascular endothelial growth factor receptor. It has exhibited broad immunomodulatory potential by promoting vascular normalization, facilitating T cell infiltration, and downregulating PD‐L1 expression in the TME, collectively fostering an immunosupportive TME [25, 26, 27, 28]. Together, these features position niraparib plus anlotinib as a compelling candidate bridging therapy to potentiate CAR‐T cell therapy in ovarian cancer.

In this study, we investigated PARP inhibition plus anlotinib as a bridging therapy prior to the infusion of transforming growth factor β (TGFβ)‐resistant, dual‐target mesothelin (MSLN)/CD19 CAR‐T cells in preclinical models of ovarian cancer. We examined whether this temporally separated regimen induced durable TME remodeling and enhanced subsequent CAR‐T cell infiltration and antitumor activity.

## 2 Results

### 2.1 PARP Inhibitor Combined With Anlotinib Promotes Durable T Cell Infiltration

PARP inhibitors and anlotinib exert immunostimulatory effects that can enhance T cell infiltration within the TME [12, 25]. However, whether such immunologic remodeling persists after treatment withdrawal remains unclear. To address this question, we evaluated the durability of niraparib plus anlotinib‐induced T cell infiltration in C57BL/6 mice bearing syngeneic ID8 ovarian tumors (Figure 1A). Consistent with its reported clinical activity [23], niraparib plus anlotinib delayed tumor progression (Figure 1B,C and Figure S1A). Flow cytometry analysis demonstrated an increase in tumor‐infiltrating T cells after combination treatment (Figure 1D and Figure S1B), which was further supported by immunohistochemical staining for CD4^+^ and CD8^+^ cells (Figure 1E,F). Compared with vehicle‐treated controls, the combination group showed 3.37‐fold and 4.46‐fold higher levels of CD4^+^ and CD8^+^ T cell infiltration, respectively (Figure 1E,F). Notably, this increase in intratumoral T cell abundance was not transient; rather, infiltration persisted for 7 days after treatment withdrawal (Figure 1G–I) and remained evident at day 14 after treatment cessation (Figure 1J–L and Figure S1C,D). This suggested sustained immunologic reprogramming of the TME induced by the combination therapy. Moreover, the combination of niraparib and anlotinib was well tolerated, with no significant body weight loss (Figure S1E), biochemical or hematologic toxicity (Figure S1F), or histopathologic abnormalities in major organs (Figure S1G). To further examine whether durable T cell infiltration could be induced by PARP inhibition beyond niraparib, we performed supportive validation employing another clinically used PARP inhibitor, olaparib, in the syngeneic ID8 tumor model (Figure S1H). Similarly, olaparib monotherapy induced a persistent increase in intratumoral T cell infiltration (Figure S1I,J). Collectively, these findings indicated that PARP inhibition, particularly when combined with anlotinib, promoted durable T cell enrichment within the TME, providing a rationale for its use as bridging therapy to facilitate subsequent CAR‐T cell infiltration in ovarian cancer.

**FIGURE 1:**
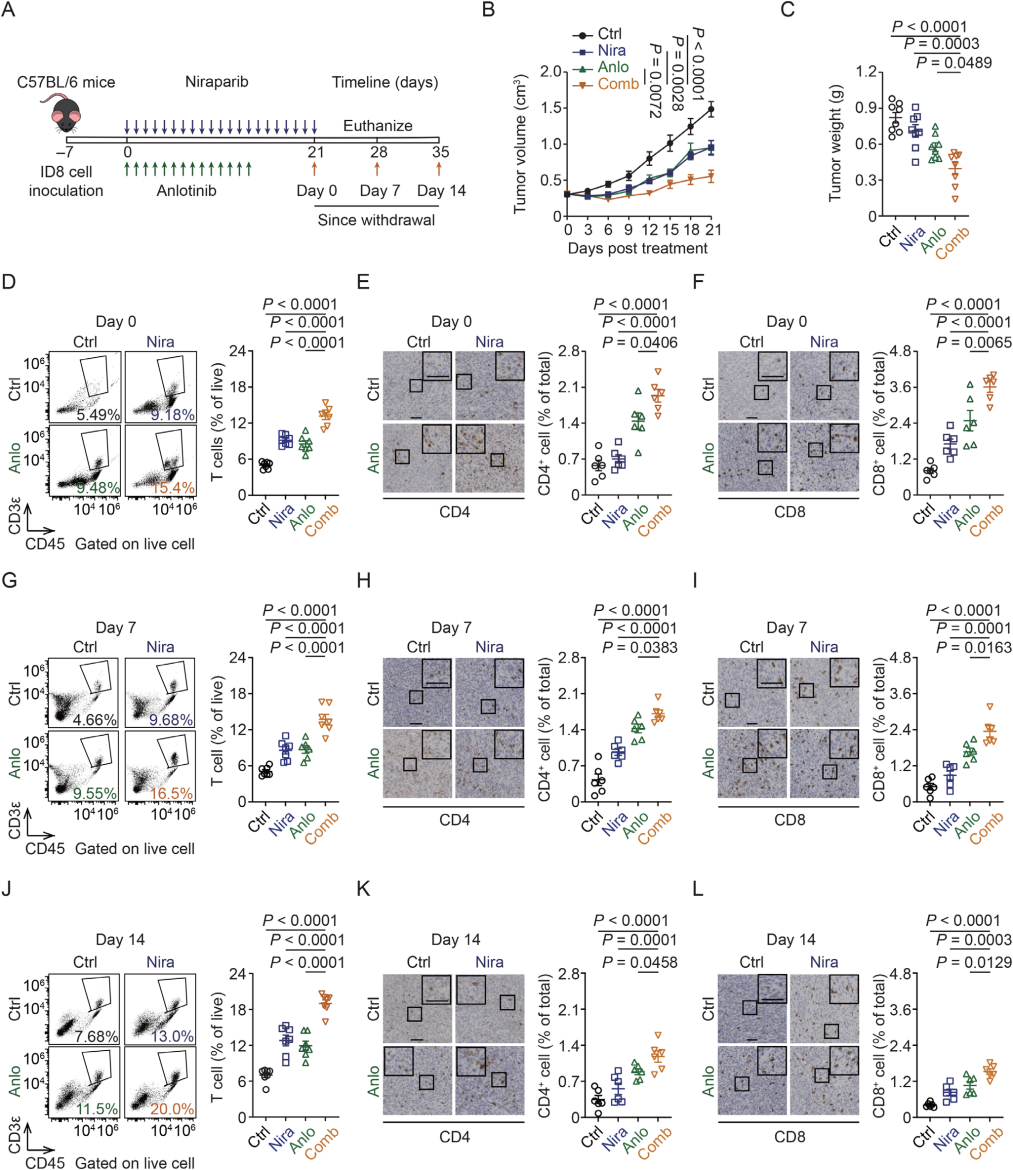
PARP inhibitor combined with anlotinib promotes durable T cell infiltration. (A) Schematic of the experimental design in ID8 tumor‐bearing mice treated with vehicle (Ctrl), niraparib (Nira), anlotinib (Anlotinib), or the combination (Comb). (B) Tumor growth curves. *n* = 10–12 biologically independent mice per group. Statistical significance was assessed by two‐way repeated‐measures analysis of variance (ANOVA). (C) Tumor weight at day 21 post treatment. *n* = 8 per group. (D, G, and J) Representative flow cytometry plots (left) and quantification (right) of tumor‐infiltrating CD3^+^ T cells among live cells at day 0 (D), day 7 (G), and day 14 (J) following treatment withdrawal. *n* = 6 or 7 per group. (E, H, and K) Representative immunohistochemical images (left) and quantification (right) of intratumoral CD4^+^ cells (brown) among total cells at day 0 (E), day 7 (H), and day 14 (K) after withdrawal. *n* = 6 or 7 per group. (F, I, and L) Representative immunohistochemical images (left) and quantification (right) of intratumoral CD8^+^ cells (brown) among total cells at day 0 (F), day 7 (I), and day 14 (L) following withdrawal. *n* = 6 per group. For C–L, statistical significance was determined using one‐way ANOVA with Tukey's post hoc multiple‐comparison test. Nuclei were counterstained with hematoxylin (blue) and boxed areas were further magnified (E, F, H, I, K, and L). Data are presented as mean ± SEM (B–L). Scale bars, 100 µm (E, F, H, I, K, and L).

### 2.2 Niraparib Plus Anlotinib Promotes TME Remodeling Permissive to T Cell Trafficking

Effective T cell infiltration into the TME requires a coordinated sequence of events, including extravasation across tumor‐associated vasculature, chemotactic guidance, and navigation through the ECM [29]. PARP inhibitors can induce DNA damage that activates the cGAS‐STING signaling axis, thereby promoting the production of chemotactic mediators and enhancing T cell recruitment in a STING‐dependent manner [12]. Using the treatment‐withdrawal model described above (Figure 1A), we found that niraparib plus anlotinib increased the γH2AX signal within the TME, with levels 7.18‐fold higher than those observed in vehicle‐treated controls (Figure S2A). Although the γH2AX signal gradually declined after treatment cessation, it remained elevated in the combination group compared with controls or monotherapies at both 7 and 14 days after treatment withdrawal (Figure S2B and Figure 2A). This residual DNA damage was associated with sustained phosphorylation of TBK1 (Figure 2B and Figure S2C,D), a key downstream event of cGAS‐STING pathway activation. Consistent with this signaling response, niraparib plus anlotinib increased the level of downstream chemotactic mediators, including C‐X‐C motif chemokine ligand 10 (CXCL10) (Figure 2C and Figure S2E,F), a key mediator of T cell recruitment, and C‐C motif chemokine ligand 5 (CCL5) (Figure S2G–I). These findings suggested that niraparib plus anlotinib activated cGAS‐STING‐associated chemotactic signaling, which may contribute to T cell infiltration.

**FIGURE 2:**
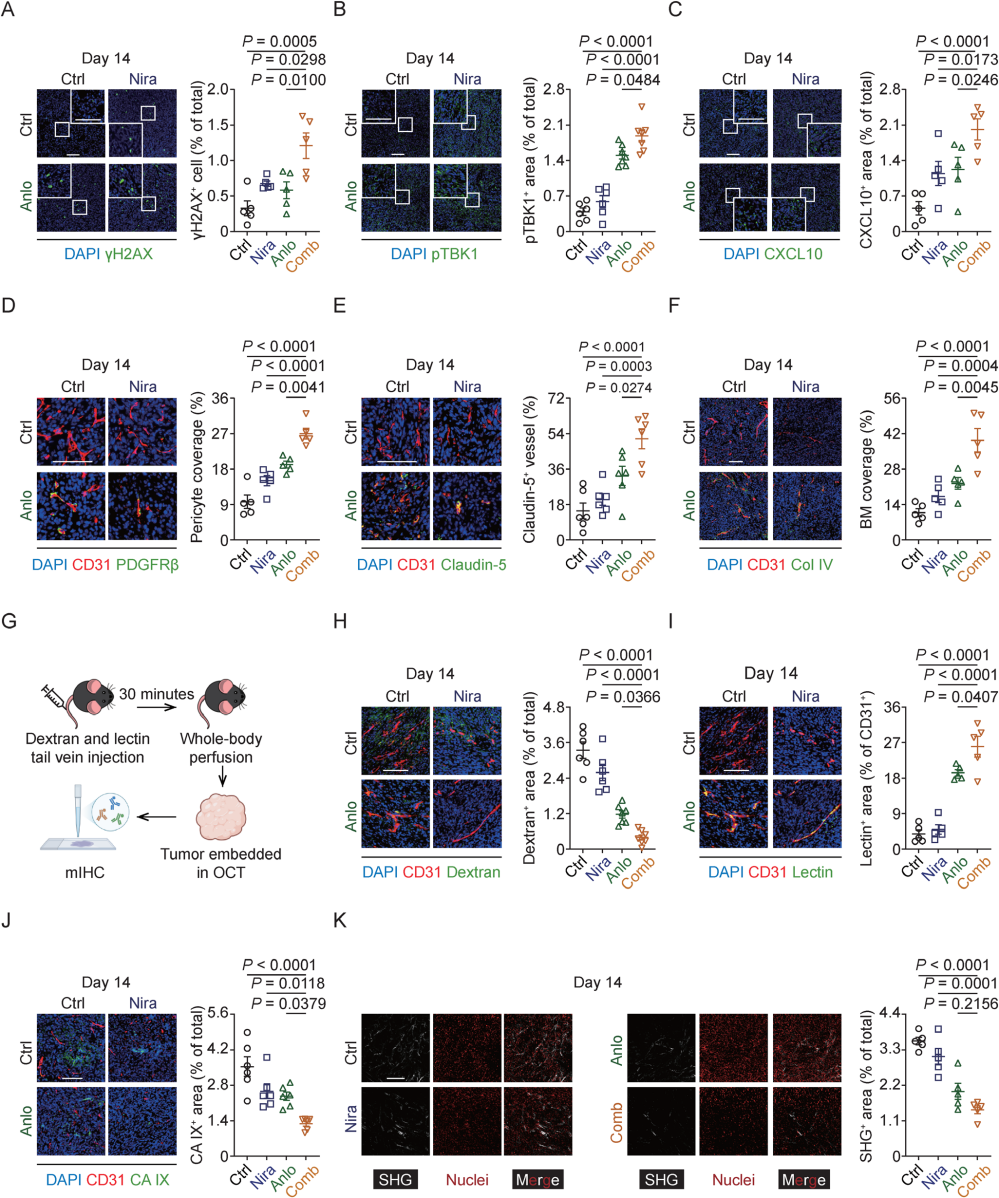
Niraparib plus anlotinib promotes TME remodeling permissive to T cell trafficking. (A–C) Representative immunofluorescence staining (left) and quantification (right) of γH2AX (A), pTBK1 (B), and CXCL10 (C) (green) in tumor sections at day 14 following treatment withdrawal. Boxed areas were further magnified. *n* = 5–7 biologically independent mice per group. Ctrl denotes vehicle; Nira denotes niraparib; Anlo denotes anlotinib; and Comb denotes the combination of niraparib and anlotinib. (D–F) Immunofluorescence co‐staining (left) of PDGFRβ (D), claudin‐5 (E), or Col IV (F) (green) with CD31 (red) and corresponding quantification (right) of pericyte coverage (D), claudin‐5^+^ vessels (E), or basement membrane (BM) integrity (F) at day 14 following withdrawal. *n* = 5 or 6 per group. (G) Experimental schematic illustrating the assessment of vascular leakage (dextran) and perfusion (lectin). mIHC, multiplex immunohistochemistry; OCT, optimal cutting temperature. The diagram was generated using BioRender. (H,I) Representative immunofluorescence images (left) and quantification (right) of vascular leakage (dextran, pseudo‐colored green, H) and perfusion (lectin, pseudo‐colored green, I) with CD31 (red) at day 14 following withdrawal. *n* = 5–7 per group. (J) Immunofluorescence co‐staining (left) of CA IX (green) with CD31 (red) and quantification (right) of CA IX^+^ hypoxia regions at day 14 following withdrawal. *n* = 6 per group. (K) Representative SHG imaging (left) and quantification (right) of fibrillar collagen fiber density at day 14 after withdrawal. Fibrillar collagen fibers are shown in white, and nuclei were stained with To‐Pro‐3 (red). *n* = 5 per group. SHG, second harmonic generation. For panels A–F and H–K, statistical significance was determined using one‐way analysis of variance (ANOVA) followed by Tukey's post hoc multiple‐comparison test. Nuclei were counterstained with DAPI (blue) for A–F and H–J. Data are presented as mean ± SEM (A–F and H–K). Scale bars, 100 µm (A–F and H–K).

Tumor‐associated vasculature is frequently disorganized and dysfunctional, with a tortuous architecture, reduced pericyte coverage, discontinuous basement membrane structure, and impaired endothelial junctions. These abnormalities contribute to hypoperfusion, hypoxia, and a TME poorly permissive to T cell extravasation [30]. Niraparib plus anlotinib reduced overall vascular density (Figure S3A–C) while promoting features associated with vessel normalization. Specifically, the combination treatment increased platelet‐derived growth factor receptor β^+^ (PDGFRβ^+^) pericyte coverage (Figure 2D and Figure S3D,E), enhanced localization of the endothelial junctional molecule claudin‐5 (Figure 2E and Figure S3F,G), and improved collagen IV^+^ (Col IV^+^) basement membrane continuity compared with controls and monotherapies (Figure 2F and Figure S3H,I). Using dextran and lectin assays to assess vascular leakage and perfusion, respectively (Figure 2G), we found that niraparib plus anlotinib reduced vascular leakage (Figure 2H and Figure S3J,K) and improved perfusion (Figure 2I and Figure S3L,M). These structural and functional vascular changes were accompanied by a reduction in CA IX staining (Figure 2J and Figure S3N,O), with the combination treatment decreasing CA IX‐positive signal by 47.8% relative to vehicle‐treated controls at 14 days after treatment withdrawal (Figure 2J), suggesting partial attenuation of intratumoral hypoxia. Notably, several features of vascular normalization persisted for up to 14 days after treatment cessation, suggesting durable vascular remodeling characterized by improved endothelial integrity, basement membrane organization, and perfusion, which may collectively facilitate T cell trafficking.

The dense fibrotic ECM within the TME represents a physical barrier that restricts T cell motility and infiltration [31]. Collagens are principal structural components of the ECM [32]. Second harmonic generation (SHG) imaging revealed that niraparib plus anlotinib reduced fibrillar collagen fiber density, an effect that persisted for at least 14 days after treatment discontinuation (Figure 2K and Figure S4A,B). Anlotinib largely accounted for this antifibrotic effect, which was further corroborated by complementary histologic analyses. These included Masson's trichrome (Figure S4C–E) and picro‐sirius red staining (Figure S4F–H), both of which demonstrated reduced fibrillar collagen deposition. Such ECM remodeling may facilitate T cell infiltration by relieving physical constraints on cell migration and supporting vascular decompression and perfusion.

Cytokine array profiling further indicated that niraparib plus anlotinib reshaped the tumor cytokine milieu (Figure S5A–C). The increased TGFβ1 level highlighted the immunosuppressive pressure that T cells may encounter within the remodeled TME. In parallel, cytokines including interleukin (IL)‐4, IL‐12 p70, IL‐21, and IL‐28 were also modulated by bridging therapy. These cytokines have been implicated in the regulation of T cell proliferation, activation, effector function, and exhaustion [33, 34, 35, 36]; in particular, IL‐12 p70, IL‐28, and interferon γ (IFNγ) may further facilitate T cell infiltration through activation of immunostimulatory and chemokine‐related pathways [37, 38, 39]. Together, these findings suggested that niraparib plus anlotinib induced coordinated and durable remodeling of the TME.

### 2.3 Engineering TGFβ‐Resistant MSLN/CD19 Dual‐Target CAR‐T Cells Using a Bait‐and‐Switch Strategy

MSLN is a cell‐surface glycoprotein aberrantly overexpressed in a broad spectrum of solid tumors, including ovarian cancer, and represents an attractive therapeutic target for CAR‐T cell therapies [40]. To enhance the in vivo expansion, persistence, and functional fitness of MSLN‐directed CAR‐T cells, we adopted a bait‐and‐switch design by incorporating an auxiliary CD19‐targeting CAR [41]. Because CD19 is abundantly expressed on normal B cells, the auxiliary CD19‐CAR is designed to provide antigen‐driven stimulation in the peripheral circulation, thereby supporting CAR‐T cell activation, proliferation, and persistence before subsequent engagement of MSLN‐expressing tumor cells within the TME (Figure 3A and Figure S6A). In addition, given the immunosuppressive effects of TGFβ and the increased TGFβ1 signal observed within the cytokine milieu induced by niraparib plus anlotinib (Figure S5), we co‐expressed a dominant‐negative TGFβ Receptor II (dnTGFβRII) in CAR‐T cells. This modification is expected to abrogate TGFβ signaling and confer resistance to TGFβ‐mediated suppression (Figure 3A) [42].

**FIGURE 3:**
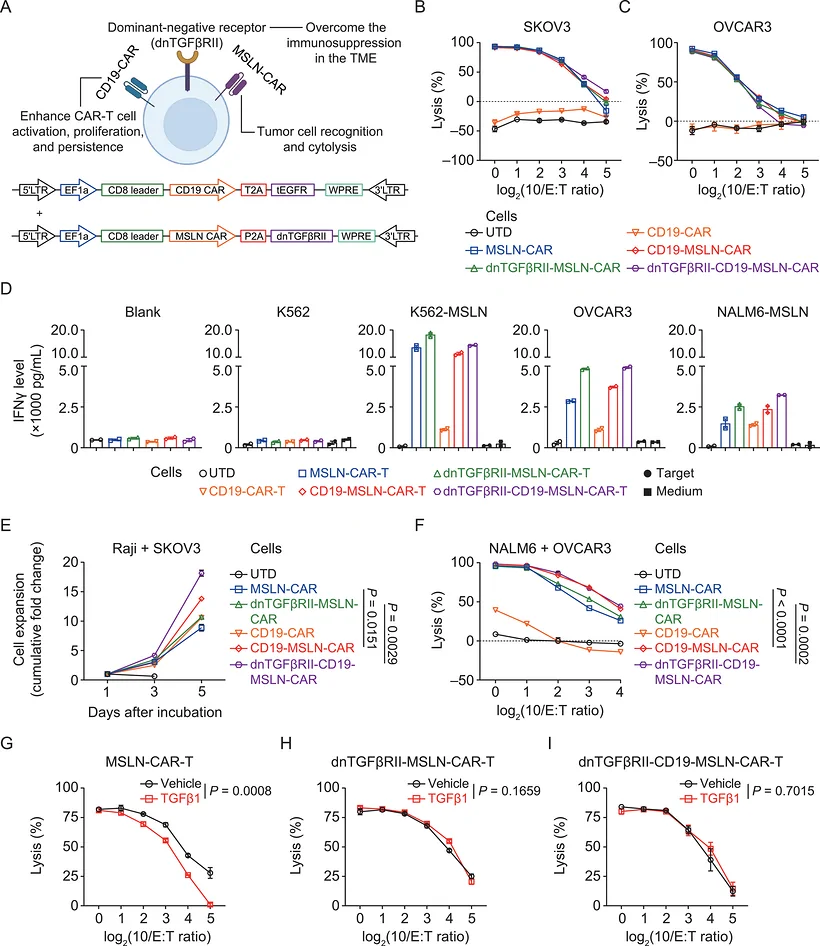
Engineering TGFβ‐resistant MSLN/CD19 dual‐target CAR‐T cells using a bait‐and‐switch strategy. (A) Schematic of CAR‐T cell architecture. The first CAR (top) targeted CD19 and incorporated a truncated EGFR (tEGFR) safety switch. The second CAR (bottom) recognized MSLN and co‐expressed a dnTGFβRII. TME, tumor microenvironment. (B,C) Cytotoxicity of untransduced T cells (UTD) and CAR‐T cells against SKOV3 (B) and OVCAR3 (C) cells at varying effector‐to‐target (E:T) ratios. *n* = 3 technical replicates per group. (D) IFNγ secretion in co‐cultures of UTD or CAR‐T cells with blank control (column 1), K562 (column 2), MSLN‐expressing K562 (column 3), OVCAR3 (column 4), or MSLN‐expressing NALM6 (column 5) cells at an E:T ratio of 5:1. *n* = 2 technical replicates per group. (E) Expansion dynamics of UTD and CAR‐T cells during co‐culture with Raji and SKOV3 cells. *n* = 2 technical replicates per group. Statistical significance was assessed by two‐way repeated‐measures analysis of variance (ANOVA). (F) The cytotoxicity of UTD and CAR‐T cells against OVCAR3 cells at varying E:T ratios after co‐culture with NALM6 cells. *n* = 3 technical replicates per group. Statistical analysis employed a mixed‐effects model with restricted maximum likelihood (REML) estimation (red vs. blue) or two‐way repeated‐measures ANOVA (purple vs. green). (G–I) Cytotoxicity of MSLN‐CAR‐T (G), dnTGFβRII‐MSLN‐CAR‐T (H), and dnTGFβRII‐CD19‐MSLN‐CAR‐T (I) cells against SKOV3 cells in the presence or absence of exogenous TGFβ1. *n* = 3 technical replicates per group. Statistical comparisons were performed using two‐way repeated‐measures ANOVA (G and H) or a mixed‐effects model with REML estimation (I). Descriptive phenotypic validations without formal hypothesis testing for B–D. Data are presented as mean ± SEM (B–I).

We first characterized the phenotype and function of the engineered CAR‐T cell variants, including MSLN‐targeting CAR‐T cells (MSLN‐CAR‐T), its derivative expressing a dnTGFβRII (dnTGFβRII‐MSLN‐CAR‐T), CD19‐targeting CAR‐T cells (CD19‐CAR‐T), dual‐target MSLN/CD19 CAR‐T cells (CD19‐MSLN‐CAR‐T), and its dnTGFβRII‐expressing counterpart (dnTGFβRII‐CD19‐MSLN‐CAR‐T). MSLN‐CAR‐T, dnTGFβRII‐MSLN‐CAR‐T, CD19‐MSLN‐CAR‐T, and dnTGFβRII‐CD19‐MSLN‐CAR‐T cells efficiently lysed SKOV3 cells, which endogenously expressed MSLN but lacked CD19 (Figure 3B). In contrast, untransduced T cells (UTD) and CD19‐CAR‐T cells showed minimal cytotoxicity (Figure 3B). Similar findings were observed in the independent ovarian cancer cell line OVCAR3 (Figure 3C). Consistently, co‐culture with K562 cells lacking both CD19 and MSLN (negative control) did not induce evident IFNγ secretion (Figure 3D). However, co‐culture with MSLN‐expressing K562 cells or OVCAR3 cells, both serving as MSLN single‐positive targets, induced IFNγ release from all MSLN‐directed constructs (Figure 3D and Figure S6B,C). Exposure to MSLN‐expressing NALM6 cells, which endogenously expressed CD19 and were further engineered to express MSLN, elicited IFNγ production across all CAR‐T cell variants (Figure 3D and Figure S6D,E).

Functionally, CD19‐mediated stimulation provided by Raji cells enhanced the proliferative capacity of CD19‐MSLN‐CAR‐T and dnTGFβRII‐CD19‐MSLN‐CAR‐T cells upon subsequent exposure to SKOV3 cells (Figure 3E). These cells outperformed their MSLN‐CAR‐T and dnTGFβRII‐MSLN‐CAR‐T cell counterparts (Figure 3E). Consistent with this observation, under CD19 stimulation delivered by NALM6 cells, the dual‐target constructs, including CD19‐MSLN‐CAR‐T and dnTGFβRII‐CD19‐MSLN‐CAR‐T cells, showed enhanced cytotoxic activity against OVCAR3 cells (Figure 3F). In parallel, exogenous TGFβ1 suppressed the cytolytic activity of MSLN‐CAR‐T cells (Figure 3G). In contrast, dnTGFβRII‐modified variants, including dnTGFβRII‐MSLN‐CAR‐T and dnTGFβRII‐CD19‐MSLN‐CAR‐T cells, maintained cytotoxic activity (Figure 3H,I), supporting their resistance to TGFβ1‐mediated suppression.

Then, we evaluated these CAR‐T cell constructs in vivo. Administration of high‐dose MSLN‐CAR‐T, dnTGFβRII‐MSLN‐CAR‐T, CD19‐MSLN‐CAR‐T, or dnTGFβRII‐CD19‐MSLN‐CAR‐T cells induced strong tumor control in NCG mice bearing SKOV3 tumors without evident body weight loss (Figure 4A–C and Figure S7A). CAR‐T cell expansion in peripheral blood peaked at day 14 after infusion (Figure S7B). Under a low‐dose CAR‐T cell regimen supplemented with weekly infusions of replication‐deficient NALM6 cells as CD19 sources (Figure 4D), MSLN‐CAR‐T cells showed limited antitumor activity (Figure 4E,F). In contrast, MSLN‐CAR‐T cell constructs, incorporating either dnTGFβRII or the CD19‐targeting CAR, demonstrated improved tumor control (Figure 4E,F). Although dnTGFβRII‐CD19‐MSLN‐CAR‐T cells showed a trend toward greater antitumor activity compared with dnTGFβRII‐MSLN‐CAR‐T cells, this difference did not reach statistical significance (*p* = 0.0571) (Figure 4E). Across all groups, mice maintained stable body weight (Figure S7C), and CAR‐T cell expansion again peaked at day 14 after infusion, followed by gradual contraction (Figure S7D). Collectively, these data showed that dual‐target MSLN/CD19 CAR‐T cells engineered with TGFβ resistance exhibited enhanced proliferative capacity, persistence, and cytotoxicity.

**FIGURE 4:**
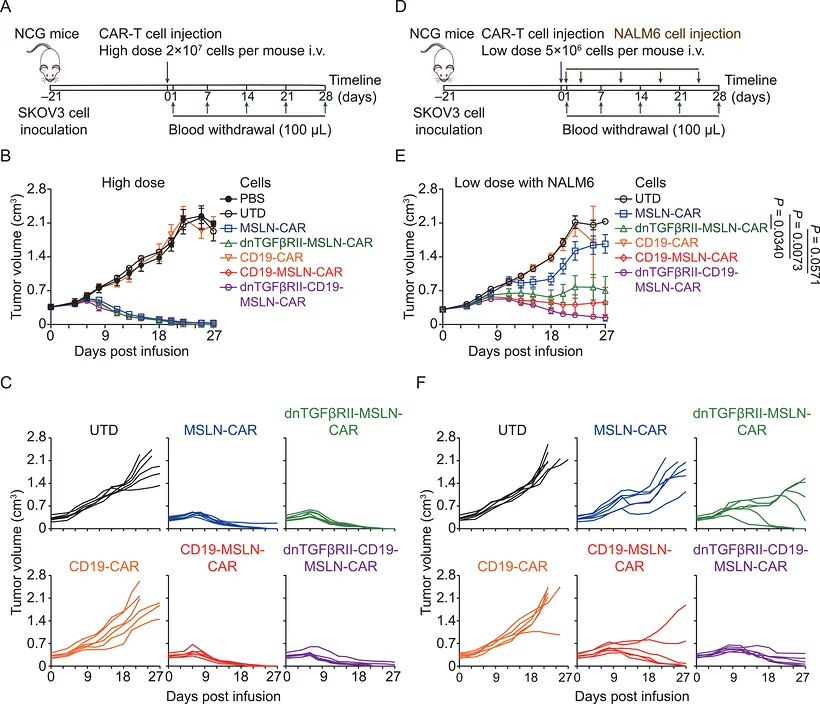
TGFβ‐resistant MSLN/CD19 dual‐target CAR‐T cells exhibit enhanced antitumor efficacy in vivo. (A) Schematic of subcutaneous SKOV3 tumor model treated with a high dose of CAR‐T cells. (B) Tumor growth plot. *n* = 6 biologically independent mice per group. UTD, untransduced T cells; PBS, phosphate‐buffered saline. (C) Individual tumor growth curves for mice administered with a high dose of UTD (top, left), MSLN‐CAR‐T (top, middle), dnTGFβRII‐MSLN‐CAR‐T (top, right), CD19‐CAR‐T (bottom, left), CD19‐MSLN‐CAR‐T (bottom, middle), or dnTGFβRII‐CD19‐MSLN‐CAR‐T (bottom, right) cells. *n* = 6 per group. (D) Experimental schema of subcutaneous SKOV3 tumor model treated with a low dose of CAR‐T cells supplemented with replication‐deficient NALM6 cells. (E) Tumor growth plot. *n* = 6 per group. Statistical analysis was performed using a mixed‐effects model with restricted maximum likelihood (REML) estimation (green vs. blue; red vs. blue) or two‐way repeated‐measures analysis of variance (ANOVA) (purple vs. green). (F) Individual tumor growth curves for mice receiving a low dose of UTD (top, left), MSLN‐CAR‐T (top, middle), dnTGFβRII‐MSLN‐CAR‐T (top, right), CD19‐CAR‐T (bottom, left), CD19‐MSLN‐CAR‐T (bottom, middle), or dnTGFβRII‐CD19‐MSLN‐CAR‐T (bottom, right) cells. *n* = 6 per group. Data are presented as mean ± SEM (B and E).

### 2.4 Bridging Therapy Potentiates CAR‐T Cell Infiltration and Antitumor Activity

Building on the engineered TGFβ‐resistant, dual‐target MSLN/CD19 CAR‐T cells, we next investigated whether niraparib plus anlotinib administered as bridging therapy could enhance CAR‐T cell infiltration and antitumor efficacy. Niraparib plus anlotinib was administered for 14 consecutive days, followed by a 7‐day washout period before CAR‐T cell infusion. Replication‐deficient NALM6 cells were injected weekly to provide a continuous source of human CD19 antigen. In NCG mice bearing subcutaneous (s.c.) OVCAR8 tumors (Figure S8A), bridging therapy followed by UTD did not significantly inhibit tumor growth compared with vehicle followed by UTD (Figure S8B,C). In contrast, bridging therapy followed by CAR‐T cell infusion achieved greater tumor suppression than vehicle followed by CAR‐T cell infusion (Figure S8B,C), supporting the interpretation that the enhanced tumor control resulted from potentiation of subsequent CAR‐T cell activity rather than from the direct antitumor effect of bridging therapy alone. Body weight remained stable across all treatment groups (Figure S8D). Consistent with these findings, in mice bearing intraperitoneal (i.p.) OVCAR8‐luci tumors (Figure S8E), bridging therapy enhanced CAR‐T cell‐mediated antitumor activity, as evidenced by lower longitudinal bioluminescence signals (Figure S8F,G) and reduced endpoint tumor burden (Figure S8H), without significant changes in body weight throughout the experiment period (Figure S8I).

Next, we evaluated this strategy in NCG mice bearing SKOV3 cell line‐derived xenograft (CDX) (Figure 5A). CAR‐T cells administered after bridging therapy showed enhanced tumor‐suppressive activity compared with vehicle followed by CAR‐T cells (Figure 5B,C and Figure S9A,B). Baseline tumor volume before CAR‐T cell infusion was comparable between groups, indicating that baseline tumor burden was not a major confounding factor affecting CAR‐T cell infiltration (Figure 5B). The differing responses to TGFβ‐resistant, MSLN/CD19 dual‐target CAR‐T cells observed between the two SKOV3 models (Figures 4E and 5B) may stem from differences in tumor burden at the time of infusion and model establishment. These differences may have contributed to the weaker antitumor activity of CAR‐T cells alone in the SKOV3 CDX model. Recent advances in radiolabeled, target‐specific positron emission tomography (PET) probes have enabled noninvasive visualization of CAR‐T cell trafficking and intratumoral activity [43]. In vivo tracking using a ^68^Ga‐labeled granzyme B (GZMB) probe showed an increased intratumoral GZMB signal 10 days after CAR‐T cell infusion in the bridging therapy group (Figure 5D), suggesting enhanced CAR‐T cell infiltration and cytotoxic activity. This finding was further supported by immunohistochemical staining for GZMB (Figure S9C). Flow cytometric analysis confirmed an increased number of tumor‐infiltrating CAR‐T cells in the bridging therapy group (Figure 5E and Figure S9D), which was corroborated by immunohistochemical staining for CD3, CD4, and CD8 (Figure 5F,G). Compared with CAR‐T cells alone, bridging therapy followed by CAR‐T cell infusion resulted in 10.70‐, 5.03‐, and 6.99‐fold higher levels of CD3^+^, CD4^+^, and CD8^+^ cells, respectively (Figure 5F,G). Importantly, this regimen was well tolerated, with no evidence of weight loss (Figure S9E), organ injury (Figure S9F,G), or hematologic toxicity (Figure S9H).

**FIGURE 5:**
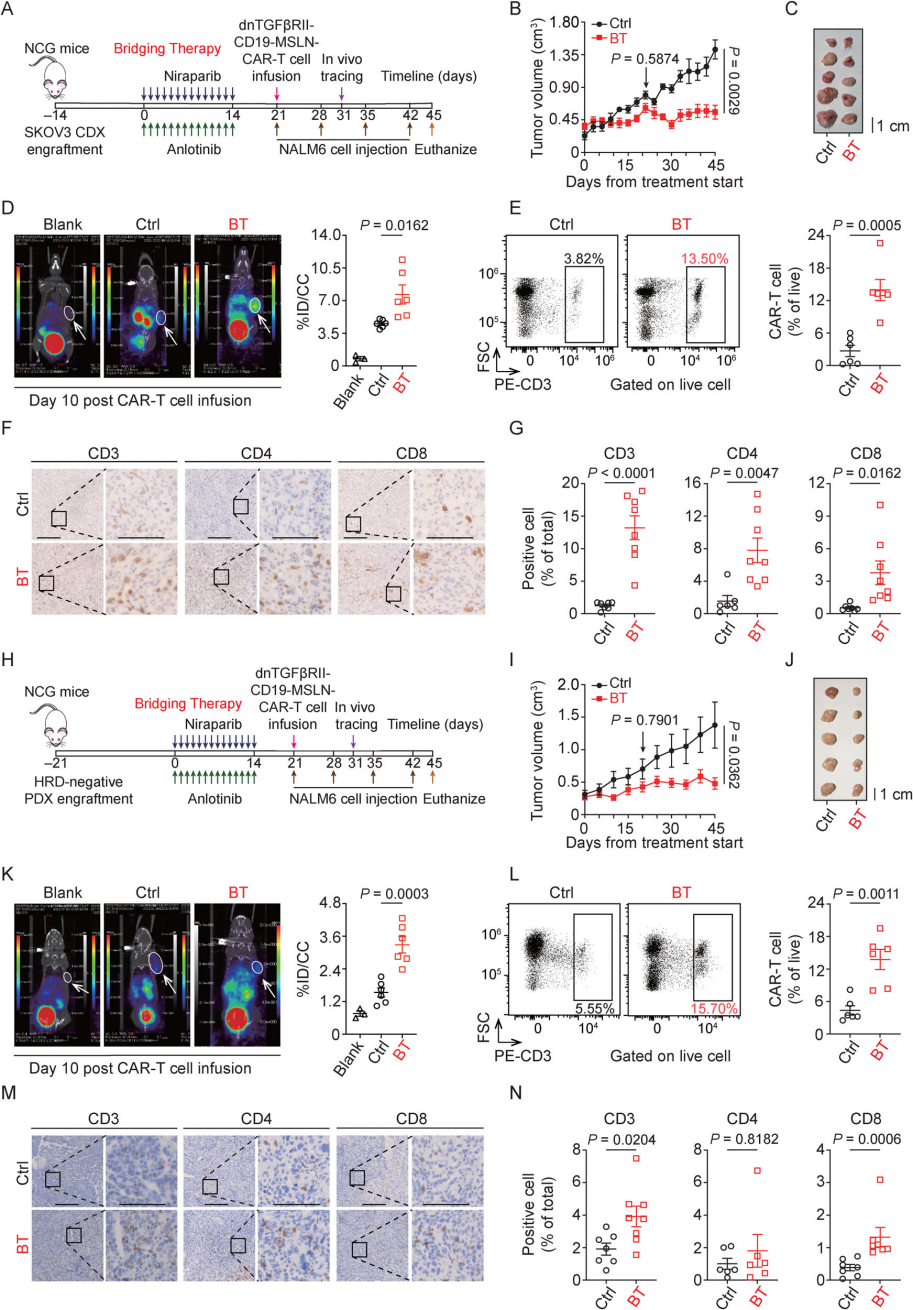
Bridging therapy potentiates CAR‐T cell infiltration and antitumor activity. (A) Experimental design of the SKOV3 CDX model treated with bridging therapy or vehicle followed by CAR‐T cell infusion. CDX, cell‐derived xenograft. (B) Tumor growth plot. *n* = 7 biologically independent mice (Ctrl), *n* = 8 (BT). Statistical analysis was performed using two‐way repeated‐measures analysis of variance (ANOVA); post hoc comparisons at individual timepoints were assessed with the Šidák multiple‐comparison test. Ctrl denotes vehicle followed by CAR‐T cells, whereas BT denotes bridging therapy followed by CAR‐T cells. (C) Representative gross images of SKOV3 CDX tumors excised from Ctrl and BT groups. Scale bar, 1 cm. (D) Representative PET‐CT images (left) and quantification (right) of intratumoral human GZMB abundance at day 10 post‐CAR‐T cell infusion. Tumor regions are indicated by white ellipses (arrows). *n* = 3 (Blank), *n* = 6 (Ctrl), *n* = 6 (BT). Statistical significance was determined by one‐way ANOVA with Tukey's post hoc multiple‐comparison test. Blank denotes a lack of CAR‐T cell infusion. (E) Flow cytometry plots (left) and quantification (right) of tumor‐infiltrating CAR‐T cells among live cells. *n* = 6 per group. Statistical analysis was performed using a two‐tailed unpaired Student's *t*‐test. (F,G) Representative immunohistochemical images (F) and quantification (G) of intratumoral CD3^+^ (left), CD4^+^ (middle), and CD8^+^ (right) (brown) cell infiltration. Nuclei were counterstained with hematoxylin (blue), and boxed areas were further magnified. *n* = 7 (Ctrl), *n* = 8 (BT). Statistical significance was determined using a two‐tailed unpaired Student's *t*‐test or Mann–Whitney *U*‐test. (H) Experimental design of the treatment‐naïve HRD‐negative PDX model. HRD, homologous recombination deficiency; PDX, patient‐derived xenograft. (I) Tumor growth plot. *n* = 7 (Ctrl), *n* = 10 (BT). Data were analyzed using a mixed‐effects model with restricted maximum likelihood (REML) estimation; post hoc comparisons at individual timepoints were assessed with Šidák multiple‐comparison test. (J) Representative gross images of PDX tumors excised from Ctrl and BT groups. Scale bar, 1 cm. (K) Representative PET‐CT images (left) and quantification (right) of intratumoral human GZMB abundance at day 10 post‐CAR‐T cell infusion. White ellipses marked by arrows indicate tumor regions. *n* = 3 (Blank), *n* = 6 (Ctrl), *n* = 6 (BT). Statistical significance was determined by one‐way ANOVA with Tukey's post hoc multiple‐comparison test. (L) Flow cytometry plots (left) and quantification (right) of tumor‐infiltrating CAR‐T cells among live cells. *n* = 6 per group. Statistical analysis was performed using a two‐tailed unpaired Student's *t*‐test. (M,N) Representative immunohistochemical images (M) and quantification (N) of intratumoral CD3^+^ (left), CD4^+^ (middle), and CD8^+^ (right) (brown) cell infiltration. Nuclei were counterstained with hematoxylin (blue), and boxed areas were further magnified. *n* = 6–8 per group. Statistical significance was determined using a two‐tailed unpaired Student's *t*‐test or Mann–Whitney *U*‐test. Data are presented as mean ± SEM (B, D, E, G, I, K, L, and N). Scale bars, 100 µm (F and M).

We further evaluated this strategy in a treatment‐naïve, HRD‐negative ovarian cancer patient‐derived xenograft (PDX) model (Figure 5H). Unlike the significant tumor growth inhibition observed in immunocompetent mice bearing ID8 tumors treated with niraparib plus anlotinib (Figure 1B), pretreatment with this combination in this HRD‐negative PDX model did not induce significant tumor regression (Figure 5I). This difference may be partly attributable to the shorter duration of niraparib administration and the absence of an intact immune system in NCG mice. However, consistent with the findings in the SKOV3 CDX model, bridging therapy enhanced TGFβ‐resistant, dual‐target MSLN/CD19 CAR‐T cell‐mediated tumor suppression compared with CAR‐T cells alone (Figure 5I,J and Figure S9I,J). In vivo PET imaging revealed increased intratumoral accumulation of GZMB signal in the bridging therapy group (Figure 5K), which was supported by immunohistochemical staining for GZMB (Figure S9K). Flow cytometry confirmed an increase in intratumoral CAR‐T cell abundance (Figure 5L), and immunohistochemical staining showed elevated CD3^+^ T cell infiltration (Figure 5M,N). Although CD4^+^ cell abundance did not significantly differ between groups, CD8^+^ cell infiltration increased following bridging therapy (Figure 5M,N). This regimen also demonstrated an acceptable safety profile, with only minor body weight loss that was not statistically significant (Figure S9L). Transcriptomic profiling of tumors harvested at the time of CAR‐T cell infusion indicated that bridging therapy upregulated IFN‐mediated signaling pathways and downregulated pathways associated with hypoxia and ECM assembly (Figure S9M), consistent with TME remodeling observed in ID8 ovarian tumor models. Collectively, these findings suggested that niraparib plus anlotinib as bridging therapy enhanced CAR‐T cell infiltration and antitumor activity in ovarian cancer CDX and PDX models.

### 2.5 Translational Relevance of Bridging Therapy in a Clinically Representative Ovarian Cancer Model

Advanced ovarian cancer frequently develops resistance to multiple therapeutic regimens after successive lines of treatment [44]. To better model this clinical scenario, we next assessed whether bridging therapy could enhance CAR‐T cell infiltration and antitumor activity in a *BRCA*‐mutated ovarian cancer PDX model that had experienced prior treatment (Figure 6A). The patient donor for this model had previously received two courses of standard platinum‐based chemotherapy and olaparib maintenance therapy before undergoing secondary debulking surgery for recurrent disease (Figure 6B). In this heavily pretreated setting, bridging therapy followed by TGFβ‐resistant, dual‐target MSLN/CD19 CAR‐T cell infusion suppressed tumor growth compared with CAR‐T cells alone (Figure 6C,D and Figure S10A). Serial in vivo functional imaging demonstrated sustained enrichment of intratumoral GZMB signals 10 and 20 days after CAR‐T cell infusion in the bridging therapy group (Figure 6E,F), indicating enhanced and persistent CAR‐T cell‐associated cytotoxic activity. This finding was further corroborated by endpoint immunohistochemical analysis, which showed increased intratumoral GZMB staining in the bridging therapy group (Figure S10B,C). Importantly, bridging therapy did not alter MSLN level, the target antigen of CAR‐T cells (Figure S10B,C), ruling out antigen modulation as a major confounding factor. Body weight loss in the bridging therapy group was mild and reversible (Figure S10D). Flow cytometric analyses of tumor samples collected at day 45 showed increased tumor‐infiltrating CAR‐T cells after bridging therapy (Figure 6G and Figure S10E). Consistently, tissue staining demonstrated increased infiltration of CD3^+^, CD4^+^, and CD8^+^ T cells (Figure 6H,I). Compared with vehicle followed by CAR‐T cells, bridging therapy followed by CAR‐T cell infusion resulted in 2.59‐, 3.86‐, and 5.40‐fold higher levels of CD3^+^, CD4^+^, and CD8^+^ cells, respectively (Figure 6H,I). Additionally, increased CAR‐T cell abundance was detected in the spleen (Figure S10F), suggesting improved systemic persistence.

**FIGURE 6:**
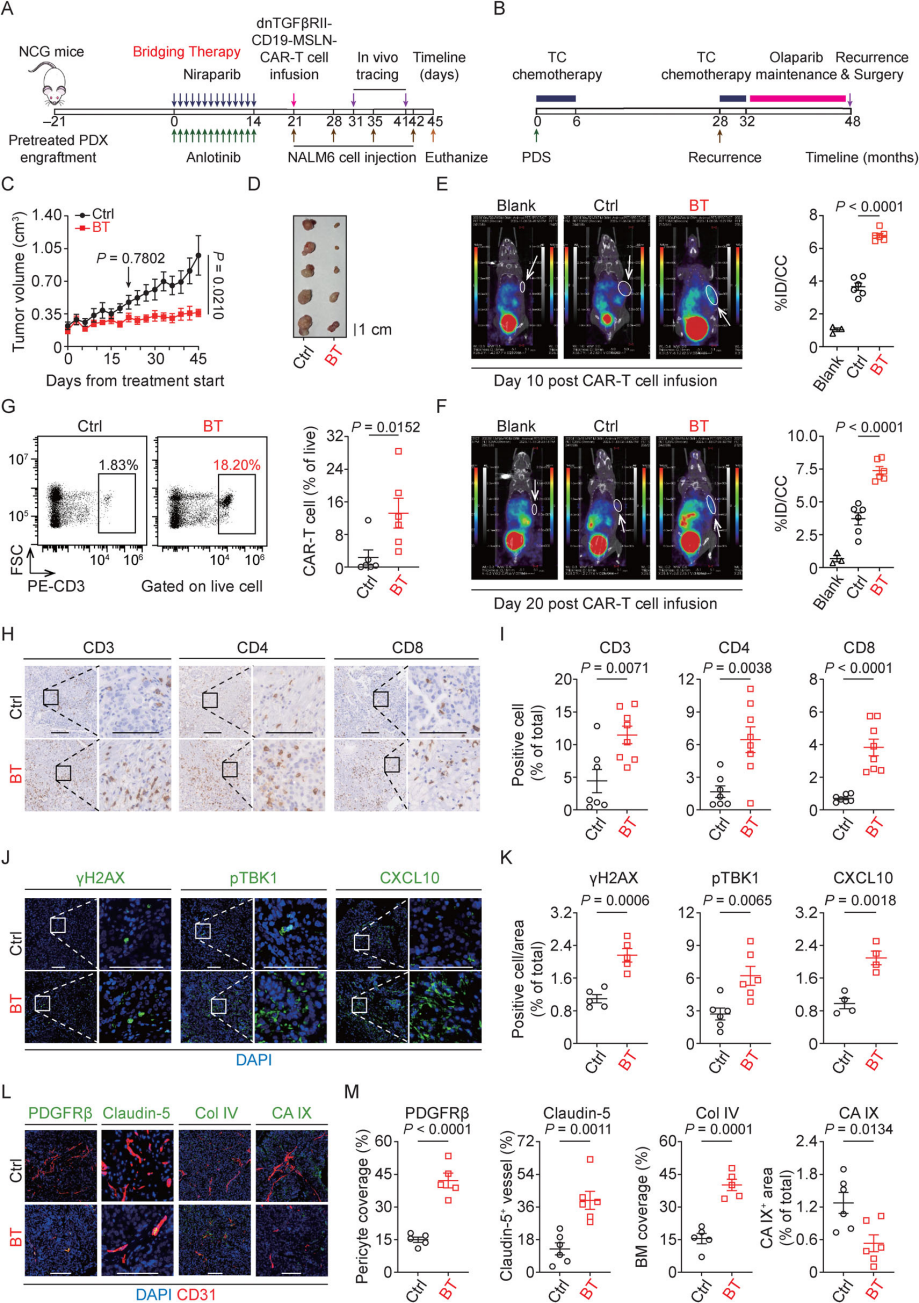
Translational relevance of bridging therapy in a clinically representative ovarian cancer model. (A) Mouse experimental design of the treatment‐experienced PDX model receiving bridging therapy followed by CAR‐T cells. PDX, patient‐derived xenograft. (B) Treatment history of the patient. TC, paclitaxel and carboplatin; PDS, primary debulking surgery. (C) Tumor growth plot. *n* = 7 biologically independent mice (Ctrl), *n* = 8 (BT). Statistical analysis was performed using two‐way repeated‐measures analysis of variance (ANOVA); post hoc comparisons at individual timepoints were assessed with the Šidák multiple‐comparison test. Ctrl denotes vehicle followed by CAR‐T cells, whereas BT denotes bridging therapy followed by CAR‐T cells. (D) Representative images of PDX tumors excised from Ctrl and BT groups. Scale bar, 1 cm. (E,F) Representative PET‐CT images (left) and quantification (right) showing intratumoral human GZMB abundance at day 10 (E) and day 20 (F) post‐CAR‐T cell infusion. White ellipses marked by arrows indicate tumor regions. *n* = 3 (Blank), *n* = 6 (Ctrl), *n* = 6 (BT). Statistical significance was determined by one‐way ANOVA. Post hoc comparisons were assessed using Tukey's multiple‐comparison test. Blank denotes a lack of CAR‐T cell infusion. (G) Flow cytometry plots (left) and quantification (right) of tumor‐infiltrating CAR‐T cells among live cells. *n* = 6 per group. Statistical analysis was performed using a two‐tailed unpaired Mann–Whitney *U*‐test. (H,I). Representative immunohistochemical images (H) and quantification (I) of CD3^+^ (left), CD4^+^ (middle), and CD8^+^ (right) (brown) cell infiltration. Nuclei were counterstained with hematoxylin (blue), and boxed areas were further magnified. *n* = 7 (Ctrl), *n* = 8 (BT). (J,K) Immunofluorescence staining (J) and quantification (K) of γH2AX (left), pTBK1 (middle), and CXCL10 (right) (green) in tumor sections. Nuclei were counterstained with DAPI (blue), and boxed areas were further magnified. *n* = 4–6 per group. (L,M) Immunofluorescence co‐staining (L) of PDGFRβ (column 1), claudin‐5 (column 2), Col IV (column 3), or CA IX (column 4) (green) with CD31 (red) and quantification (M) of pericyte coverage (column 1), claudin‐5^+^ vessels (column 2), basement membrane (BM) integrity (column 3), and CA IX^+^ hypoxic areas (column 4). Nuclei were counterstained with DAPI (blue). *n* = 5 or 6 per group. For I, K, and M, statistical significance was determined using a two‐tailed unpaired Student's *t*‐test. Data are presented as mean ± SEM (C, E–G, I, K, and M). Scale bars, 100 µm (H, J, and L).

Consistent with findings from the ID8 ovarian tumor model, bridging therapy was associated with activation of the cGAS‐STING‐related signaling axis in tumor samples collected at day 45, as reflected by higher levels of γH2AX (1.97‐fold), phosphorylated TBK1 (2.30‐fold), CXCL10 (2.14‐fold), and CCL5 (3.40‐fold) compared with vehicles (Figure 6J,K and Figure S10G,H). Vascular remodeling was also evident in this heavily pretreated model. Bridging therapy reduced vascular density (Figure S10G,H), increased PDGFRβ^+^ pericyte coverage, enhanced endothelial junctional localization of claudin‐5, and improved Col IV^+^ basement membrane continuity (Figure 6L,M). These vascular changes were accompanied by a 41.7% reduction in CA IX‐positive signal, suggesting partial attenuation of intratumoral hypoxia (Figure 6L,M). Concurrently, SHG imaging showed reduced fibrillar collagen fiber deposition in the bridging therapy group (Figure S10I,J), which was further supported by Masson's trichrome and picro‐sirius red staining (Figure S10I,J). Together, these findings indicated that in a clinically relevant, heavily pretreated ovarian cancer PDX model, niraparib plus anlotinib administered as bridging therapy enhanced CAR‐T cell infiltration and antitumor activity.

### 2.6 CAR‐T Cells Expand and are Associated With Antigen Clearance Following Bridging Therapy in a Phase I Trial

In a single‐center, exploratory phase I clinical trial (NCT05141253), we evaluated the safety and preliminary activity of TGFβ‐resistant, dual‐target MSLN/CD19 CAR‐T cells and examined the feasibility of bridging therapy in patients with recurrent or refractory MSLN‐positive solid tumors. Eligible participants were permitted to receive bridging therapy during the interval between leukapheresis and CAR‐T cell infusion (Figure 7A). Here, we present preliminary findings from the first enrolled participant. Subject 01 was a 27‐year‐old woman diagnosed with MSLN‐positive borderline mucinous ovarian cystadenoma with metastatic dissemination to the bone, liver, and left orbit. The patient had experienced disease progression after multiple prior systemic regimens, including first‐line nab‐paclitaxel plus nedaplatin, second‐line doxorubicin plus ifosfamide plus bevacizumab, and third‐line gemcitabine plus carboplatin plus bevacizumab. After enrollment and leukapheresis, she received bridging therapy consisting of olaparib (150 mg orally twice daily for 4 weeks) and anlotinib (10 mg orally once daily for 2 weeks). Olaparib was chosen over niraparib for combination with anlotinib due to the patient's decreased platelet count following multiple prior chemotherapy rounds, and niraparib's association with more pronounced thrombocytopenia than olaparib [45]. One week after the final olaparib dose, the patient received CAR‐T cell infusion at a dose of 1 × 10^6^ cells/kg.

**FIGURE 7:**
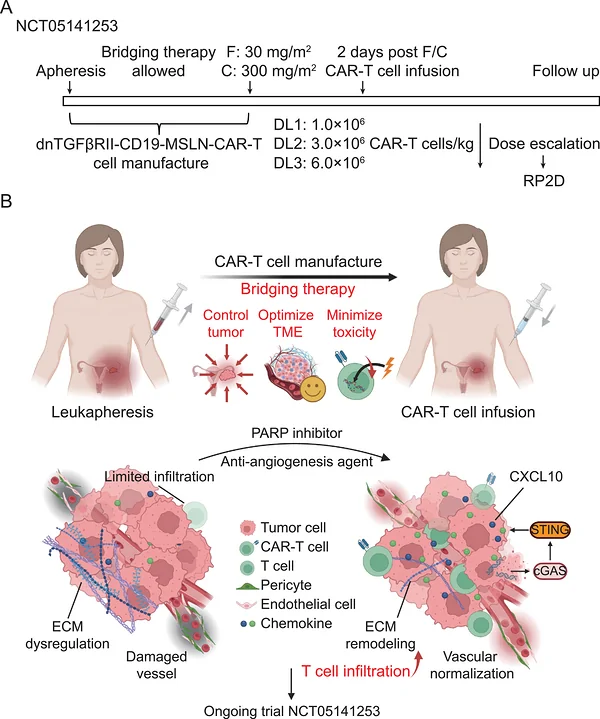
CAR‐T cells expand and are associated with antigen clearance following bridging therapy in a phase I trial. (A) Trial design of NCT05141253. F, fludarabine; C, cyclophosphamide; DL, dose level; RP2D, recommended phase 2 dose. (B) Schematic model of bridging therapy. Bridging therapy confers multiple advantages, including transient control of tumor progression, TME remodeling to enhance CAR‐T cell infiltration, and reduced direct toxicity on CAR‐T cells. The present study evaluated niraparib plus anlotinib as bridging therapy for ovarian cancer, providing rationale for a phase I clinical trial. TME, tumor microenvironment; ECM, extracellular matrix. The diagram was generated using BioRender.

The *MSLN* gene encodes a 71‐kDa precursor glycoprotein that undergoes proteolytic cleavage at arginine 295, generating a soluble 31‐kDa N‐terminal megakaryocytic potentiating factor (MPF) and a 40‐kDa membrane‐bound MSLN fragment [46]. Serial measurements showed that serum MPF concentration remained relatively stable during the first 7 days after CAR‐T cell infusion, declined sharply by day 10, and became undetectable from day 14 onward (Figure S11A), suggesting rapid clearance of MSLN‐expressing tumor cells. This kinetic pattern paralleled CAR‐T cell expansion, which began around day 7, peaked between days 10 and 14, and gradually declined thereafter (Figure S11B,C). The CD19‐targeted CAR also mediated effective B cell depletion, reducing circulating B cell counts to undetectable levels by day 10 after infusion (Figure S11D). Although this single‐patient observation lacked a control arm, these findings demonstrated in vivo CAR‐T cell expansion after bridging therapy and did not suggest that bridging therapy impaired CAR‐T cell proliferation. This trial remains ongoing, and comprehensive clinical results will be reported after study completion. Together with the preclinical data, this study provided proof‐of‐concept evidence that bridging therapy may precondition the TME and support the antitumor activity of subsequently infused CAR‐T cells in ovarian cancer (Figure 7B).

## 3 Discussion

In this study, niraparib plus anlotinib as bridging therapy enhanced CAR‐T cell infiltration and antitumor activity across multiple preclinical ovarian cancer models. This supports a mechanistically rational and clinically tractable bridging therapy with translational potential for CAR‐T cell therapy in ovarian cancer.

The journey of CAR‐T cells into the TME of solid tumors resembles a grueling stage of the Tour de France. CAR‐T cells, like powerful sprinters, possess explosive antitumor potential. However, without a lead‐out rider to shield them from headwinds and guide them through the peloton, they expend their strength prematurely and become exhausted before approaching the finish line. Progress has been made in fortifying the sprinter itself by enhancing CAR‐T cell proliferation, persistence, and cytotoxicity [47, 48, 49]. However, as in competitive cycling, success often depends on a lead‐out rider. Identifying effective lead‐out strategies that reshape the TME may provide CAR‐T cells with the necessary slipstream to reach the decisive sprint and secure therapeutic success against solid tumors.

The expanding repertoire of targeted agents with immunomodulatory properties offers attractive opportunities to serve as lead‐out riders for CAR‐T cells [8, 50]. However, direct co‐administration of these agents can exert cytotoxicity against CAR‐T cells, necessitating additional genetic modifications to generate drug‐resistant CAR‐T products [14, 51, 52]. Bridging therapy provides a conceptually distinct approach to balance immunostimulatory benefit and toxicity by temporally separating pharmacologic TME preconditioning from CAR‐T cell infusion. Clinical studies in large B‐cell lymphoma have linked baseline TME features to CAR‐T cell efficacy and safety [16, 17, 18, 20], and preclinical studies in prostate and pancreatic cancers demonstrated that cyclophosphamide conditioning can remodel the TME to improve subsequent CAR‐T cell performance [53]. Importantly, bridging therapy may be extended to other solid tumors using clinically approved drugs, tailored to individual tumor and patient characteristics. Compared with the resource‐ and time‐intensive development of drug‐resistant CAR‐T cells, bridging therapy represents a potentially faster, more cost‐effective, and clinically tractable route to translation.

The barriers limiting CAR‐T cell efficacy in solid tumors are multifaceted [7], and overcoming them requires coordinated rather than isolated interventions. In this study, we adopted an integrated design encompassing three layers of optimization. First, a dual‐target MSLN/CD19 bait‐and‐switch CAR‐T cell architecture ensured robust expansion in the circulation before antigen engagement [41]. Second, incorporation of a dnTGFβRII construct, which reduces systemic TGFβ levels [42], conferred resistance to TGFβ‐mediated suppression. Third, niraparib plus anlotinib as bridging therapy remodeled the TME to facilitate CAR‐T cell infiltration. These complementary approaches acted in concert to enhance CAR‐T cell activity.

Although synthetic lethality in *BRCA*‐mutated or HRD‐positive tumor cells represents the canonical mechanism of PARP inhibition [54, 55], the immunomodulatory effects of PARP inhibitors and their contribution to antitumor efficacy are increasingly recognized [12, 13], providing a rationale for evaluating PARP inhibitors as TME‐modulating agents even in HRD‐negative models [11, 12, 56, 57]. The combination of PARP inhibitors and antiangiogenic agents has attracted interest, as these treatments exhibit largely non‐overlapping toxicities and may exert complementary antitumor effects [58]. Niraparib plus anlotinib was selected as bridging therapy based on its reported clinical activity in platinum‐resistant recurrent ovarian cancer and the established immunomodulatory properties of both agents [11, 23, 25]. Although bridging therapy‐only groups were not included in the SKOV3 CDX and PDX experiments, the increased CAR‐T cell infiltration and GZMB activity observed after bridging therapy, together with the absence of significant tumor inhibition by bridging therapy alone in the s.c. OVCAR8 model, support the interpretation that niraparib plus anlotinib primarily acts as a TME‐modulating pretreatment that enhances subsequent CAR‐T cell efficacy rather than as a standalone antitumor treatment in these models. Our observations are consistent with previous reports that anlotinib can activate the cGAS‐STING axis or upregulate CCL5 to recruit T cells [27, 59], and can reduce ECM stiffness via inhibiting the RhoA/ROCK signaling cascade [60]. PARP inhibition also suppresses angiogenesis [61]. The immunoregulatory effects of this combination were further supported by cytokine array analysis, which indicated remodeling of the tumor cytokine milieu. However, because the cytokine analysis involved a limited sample size and exploratory unadjusted statistical comparisons, these data should be interpreted cautiously.

This study has certain limitations. First, although both s.c. and i.p. tumor models were evaluated, detailed analyses of T cell infiltration were conducted predominantly in s.c. tumors. Given that ovarian cancer typically manifests as disseminated i.p. disease with extensive metastases and ascites, further studies utilizing i.p. or orthotopic tumor models are warranted to validate these findings within the peritoneal TME. Second, B cells within the TME can support T cell responses [62]. Therefore, although the dual‐target MSLN/CD19 design enhanced expansion, it might also deplete intratumoral B cells. Further investigation is needed to determine the net effect on antitumor immunity. Third, although the use of human CAR‐T cells in highly immunodeficient mice improved translational relevance, these models do not fully recapitulate the complex interactions between CAR‐T cells and an intact host immune system. This limitation is particularly relevant because niraparib plus anlotinib was intended to remodel the TME prior to CAR‐T cell infusion. Future studies using syngeneic immunocompetent models and murine CAR‐T cells are warranted to further validate the CAR‐T cell‐recruiting effects of bridging therapy and the efficacy of the bait‐and‐switch strategy in an intact immune context. Finally, although preliminary observations from the ongoing phase I trial are encouraging, comprehensive safety and efficacy data will be reported upon formal trial completion and are required to determine clinical value.

## 4 Conclusion

In conclusion, bridging therapy with niraparib plus anlotinib enhanced CAR‐T cell infiltration and antitumor efficacy in preclinical ovarian cancer models. This supports bridging therapy as a clinically translatable TME‐preconditioning strategy to potentiate CAR‐T cell therapy in solid tumors.

## 5 Methods

### 5.1 Cell Lines and Culture

The ID8 cell line was generously provided by Professor K. Roby (Department of Anatomy and Cell Biology, University of Kansas, Lawrence, KS, USA). SKOV3 (HTB‐77, CVCL_0532), OVCAR3 (HTB‐161, CVCL_0465), Raji (CCL‐86, CVCL_0511), NALM6 (CRL‐3273, CVCL_UJ05), and K562 (CCL‐243, CVCL_0004) cell lines were obtained from the American Type Culture Collection (Manassas, VA, USA). OVCAR8 (CVCL_1629) cells were obtained from a laboratory‐maintained cell stock. MSLN‐expressing K562 and NALM6 cells were kindly provided by IASO BioTherapeutics (Nanjing, China). These cells had been generated by lentiviral transduction and subsequently enriched through puromycin selection and fluorescence‐activated cell sorting. All cell lines were routinely tested for mycoplasma contamination and authenticated by short tandem repeat profiling.

Engineered CAR‐T cell products, including MSLN‐CAR‐T, dnTGFβRII‐MSLN‐CAR‐T, CD19‐CAR‐T, CD19‐MSLN‐CAR‐T, and dnTGFβRII‐CD19‐MSLN‐CAR‐T cells, were generated and supplied by IASO BioTherapeutics using the lentiviral constructs described in patent WO2022257984A1. Approximately seven days after lentiviral transduction, expression of MSLN‐CAR, CD19‐CAR, and dnTGFβRII was evaluated by flow cytometry, as applicable to each construct. The corresponding marker‐positive fractions are summarized in Table S2. Detailed manufacturing and flow cytometric detection procedures are described in patent WO2022257984A1. The MSLN‐ and CD19‐targeting CAR constructs comprised antigen‐specific single‐chain variable fragments, in which the variable domains were linked via a (G4S)_3_ linker, followed by a CD8α hinge, transmembrane domain, and an intracellular signaling module containing 4‐1BB and CD3ζ domains (Figure S6A). The dnTGFβRII was constructed by truncating the human TGFβRII to remove the intracellular kinase domain, as originally described [63].

ID8 cells were cultured in Dulbecco's Modified Eagle's medium (#11965092, Gibco, Waltham, MA, USA). SKOV3 cells were maintained in McCoy's 5A medium (#16600082, Gibco). OVCAR3, OVCAR8, Raji, and NALM6 cells were cultured in Roswell Park Memorial Institute 1640 medium (#11875093, Gibco). K562 cells were cultured in Iscove's Modified Dulbecco's medium (#12440053, Gibco). Human CAR‐T cells were grown in CTS OpTmizer T cell expansion serum‐free medium (#A1048501, Gibco). All media were supplemented with 5–15% fetal bovine serum (#10091‐148, Gibco) and 1% penicillin/streptomycin solution (#G4003, ServiceBio, Wuhan, China), and cells were incubated at 37°C in a humidified atmosphere with 5% CO_2_. For CAR‐T cells, 1% L‐Glutamine (#25030081, Thermo Fisher Scientific, Waltham, MA, USA) and 15 ng/mL human IL‐2 (#200‐02‐100UG, PeproTech, Cranbury, NJ, USA) were additionally supplemented.

To generate replication‐incompetent target cells, NALM6 and Raji cells were treated with 30 mg/mL mitomycin C (#5791, AbMole BioScience, Houston, TX, USA) for 3 h, followed by extensive washing with fresh medium prior to use in subsequent experiments.

### 5.2 Mouse Models

All mice were maintained under specific pathogen‐free conditions and handled in accordance with the institutional guidelines for animal experimentation set by the Institutional Animal Care and Use Committee (IACUC). This study was approved by the IACUC of Tongji Hospital, Tongji Medical College, Huazhong University of Science and Technology and was performed in accordance with the Animal Research: Reporting of In Vivo Experiments (ARRIVE) guidelines [64]. Female C57BL/6 and NCG mice aged 4–6 weeks were purchased from GemPharmatech (Nanjing, China). Mice were housed in individually ventilated cages with free access to standard rodent chow and water, maintained on a 12‐h light‐dark cycle at 19°C–22°C and 30%–70% relative humidity.

For the establishment of s.c. ID8 tumors, 5 × 10^6^ ID8 cells were suspended in 100 µL of a 1:1 mixture of phosphate‐buffered saline (PBS, #G4202, ServiceBio) and Matrigel matrix (#354234, Corning, Corning, NY, USA) and injected into the right flank of C57BL/6 mice. Though the ID8 cell line is conventionally used for i.p. implantation, the s.c. ID8 model, as previously reported [65, 66, 67, 68, 69], was adopted to facilitate quantitative evaluation of T cell infiltration. For s.c. SKOV3 and OVCAR8 tumors, 8 × 10^6^ cells suspended in 100 µL of PBS/Matrigel (1:1, v/v) were injected into the right flank of NCG mice. OVCAR8 cells have been reported to harbor *BRCA1* promoter methylation, a molecular alteration associated with HRD [70]. To establish the i.p. dissemination model, NCG mice were intraperitoneally injected with 5 × 10^6^ OVCAR8‐luci cells suspended in 100 µL of PBS. When s.c. SKOV3 tumors and PDX models reached approximately 1 000 mm^3^, mice were euthanized, and tumors were excised and minced into small, viable fragments. For the establishment of formal SKOV3 CDX and PDX ovarian cancer models, mice were anesthetized, and the implantation site on the flank was sterilized. A 5‐mm skin incision was made using sterile surgical scissors, and an s.c. pocket was created by blunt dissection. A single viable tumor fragment was inserted into the pocket, and the incision was closed. Tumor dimensions were measured twice weekly using digital calipers, and tumor volume was calculated as length × width × width × 0.5. In the SKOV3 CDX model, to account for the antitumor activity of bridging therapy and the influence of tumor size on CAR‐T cell infiltration, the initial tumor burden in the bridging therapy group was deliberately established to exceed that of the control group, thereby ensuring comparable tumor volumes at the time of CAR‐T cell infusion. In all other tumor models, mice were randomized by tumor size prior to treatment initiation to ensure equivalent baseline tumor burdens across experimental groups.

For pharmacologic interventions in ID8 tumor models, niraparib (#S2741, Selleck Chemicals, Houston, TX, USA) was administered via oral gavage at 25 mg/kg daily for 21 days, formulated in sodium carboxymethyl cellulose (#S6703, Selleck Chemicals). Anlotinib (#S8726, Selleck Chemicals) was administered via oral gavage at 3 mg/kg daily for 14 days, diluted in 2% DMSO, 4% PEG400 (#S6705, Selleck Chemicals), 4% Tween‐80 (S6702, Selleck Chemicals), and 90% distilled water. Olaparib was administered via oral gavage at 50 mg/kg daily for 21 days, prepared in sodium carboxymethyl cellulose. The administered doses of niraparib, anlotinib, and olaparib were either equivalent to or lower than those conventionally employed in preclinical models. For s.c. and i.p. OVCAR8, SKOV3 CDX, and PDX models, niraparib plus anlotinib was administered via oral gavage at the aforementioned doses for 14 days. Mice in the treatment groups received 100 µL of the indicated drug solution, whereas mice in the control groups received an equal volume of the corresponding vehicle used for drug dissolution. During treatment, tumor volumes and body weights were measured at least twice weekly.

For adoptive T cell therapy, mice bearing s.c. SKOV3 tumors received either a high dose (2 × 10^7^ cells per mouse) or low dose (5 × 10^6^ cells) of CAR‐T cells via intravenous (i.v.) injection through the tail vein. UTDs were administered at the corresponding cell doses as controls. Blood samples were collected on days 1, 7, 14, 21, and 28 following CAR‐T cell infusion. Approximately 100 µL of blood was obtained via retro‐orbital puncture under anesthesia using sterile capillary tubes. In SKOV3 CDX and PDX models, 7 days after drug withdrawal, mice were injected i.v. with low‐dose CAR‐T cells (5 × 10^6^ cells). For s.c. and i.p. OVCAR8 models, the CAR‐T cell dose was reduced to 2.5 × 10^6^ cells per mouse. A 7‐day washout interval was selected based on the approximately 36‐hour elimination half‐life of niraparib [71]. This interval corresponds to approximately four to five elimination half‐lives, by which time most circulating parent drug would be expected to have been cleared, thereby reducing direct pharmacologic overlap with CAR‐T cell infusion [72]. CAR‐T cell doses were normalized according to the proportion of viable CAR‐positive T cells determined by flow cytometry before infusion. Where necessary, UTDs were added to equalize the total number of viable T cells administered across treatment groups. To maintain persistent antigenic stimulation (CD19), mice receiving low‐dose or reduced‐dose CAR‐T cells additionally received weekly i.v. injections of 1 × 10^6^ replication‐incompetent NALM6 cells. This CAR‐T cell‐to‐B cell ratio approximates clinically relevant conditions, aligning with commonly administered CAR‐T cell doses in humans (1 × 10^6^ –1 × 10^7^ cells/kg) relative to the estimated abundance of circulating B cells (approximately 1 × 10^6^ cells/kg) [73].

Mice were monitored at regular intervals and humanely euthanized before reaching ethical endpoints, defined as a tumor diameter exceeding 20 mm or signs of significant distress or body condition decline.

### 5.3 IHC

For immunohistochemistry (IHC) analysis, 4 µm sections were deparaffinized and rehydrated. Antigen retrieval was performed by boiling sections in antigen unmasking solution (#G1202 or #G1203, ServiceBio). Endogenous peroxidase activity was quenched by incubating sections in 3% hydrogen peroxide, followed by blocking with 5% normal goat serum. Sections were incubated overnight at 4°C with primary antibodies diluted to manufacturer‐recommended concentrations. Detailed information on all antibodies used in this study is provided in Table S1. After washing, sections were incubated with horseradish peroxidase‐conjugated secondary antibodies, visualized using DAB substrate (#G1212, ServiceBio), counterstained with hematoxylin, dehydrated, and mounted. High‐resolution whole‐slide images were acquired using a slide scanning system (#SQS‐40P, TEKSQRAY, Shenzhen, China).

### 5.4 mIHC

For multiplex IHC (mIHC) analysis, 4 µm sections underwent deparaffinization, rehydration, antigen retrieval, and blocking, followed by sequential primary and secondary antibody incubations as described for conventional IHC. After incubation with secondary antibodies, fluorophore‐labeled tyramide was applied using a five‐color fluorescence detection kit (RC0086‐45, RecordBio, Shanghai, China) in accordance with the manufacturer's protocol. Sequential rounds of antigen retrieval and tyramide signal amplification were performed to enable multi‐antigen labeling. Nuclei were counterstained with DAPI. Because human stromal cells are rapidly replaced by mouse stromal components in xenografted human tumors [74], identical antibodies were used for xenograft ovarian tumor sections. Fluorescent images were acquired using a digital slide scanner (Pannoramic SCAN, 3DHISTECH, Budapest, Hungary). Quantitative analyses included calculation of perivascular PDGFRβ^+^ pericyte and Col IV^+^ basement membrane coverage, and assessment of tight junction integrity based on the proportion of claudin‐5^+^ signal along CD31^+^ blood vessels. Vascular leakage and hypoxia regions were quantified as the percentage of dextran^+^ and CA IX^+^ areas relative to the total region of interest (ROI), respectively. Perfused vascular area was determined as the ratio of lectin^+^ area to CD31^+^ area.

### 5.5 Flow Cytometry

Tumor tissues were excised and finely minced with scissors into small fragments. Tissue fragments were enzymatically dissociated using the Mouse and Human Tumor Dissociation Kits (#130‐096‐730 and #130‐095‐929, Miltenyi Biotec, Bergisch Gladbach, Germany) at 37°C for 60 min on a 150‐rpm orbital shaker. The digested material was filtered through a 70 µm cell strainer to obtain single‐cell suspensions. For PDX ovarian tumor‐bearing mice, spleens were harvested, mechanically dissociated through a 40 µm cell strainer, and subjected to red blood cell lysis to generate single‐cell suspensions. To quantify CAR‐T cells in murine and human peripheral blood, samples were collected into 50 mL Falcon tubes and subjected to red blood cell lysis. The resulting single‐cell suspensions were washed with PBS, stained with a Zombie Aqua Fixable Viability Kit (#423102, BioLegend, San Diego, CA, USA) to exclude non‐viable cells, and incubated with mouse or human TruStain FcX reagents to block Fc receptor‐mediated nonspecific binding. For surface staining, cells were incubated for 30 min in the dark with fluorochrome‐conjugated antibodies diluted in staining buffer at manufacturer‐recommended concentrations. The following antibodies (Table S1) or recombinant proteins were used: FITC‐labeled human MSLN (MSN‐HF2H3, ACRO Biosystems, Newark, DE, USA) and PE‐labeled human CD19 (CD9‐HP2H5, ACRO Biosystems). Data acquisition was performed on a CytoFLEX flow cytometer (#A00‐1‐1102, Beckman Coulter, Brea, CA, USA), and data analysis was conducted using FlowJo software (v.10.8.1, BD Biosciences, Franklin Lakes, NJ, USA).

### 5.6 In Vivo CAR‐T Cell Tracking

GZMB is a serine protease secreted by cytotoxic lymphocytes, including CAR‐T cells, upon recognition of target tumor cells [75], serving as a direct biomarker of cytolytic activity. Therefore, intratumoral GZMB accumulation was noninvasively assessed to monitor CAR‐T cell infiltration and effector function in vivo, using a ^68^Ga‐labeled GZMB‐specific probe in conjunction with PET/computed tomography (CT) imaging. Mice were intravenously injected with 37 MBq of ^68^Ga‐NOTA‐GZP. One hour post injection, mice were anesthetized and PET data were acquired over a five‐minute static scan, immediately followed by a 10‐minute CT scan for anatomic reference using a small‐animal PET/CT system. Image reconstruction was performed with a three‐dimensional ordered‐subsets expectation maximization algorithm.

For quantitative analysis, tumor ROIs were delineated using NMSoft‐AIWS software (v.1.7). Specifically, representative two‐dimensional ROIs were manually drawn on the transaxial slice exhibiting the maximum tumor cross‐section. The boundaries of the ROIs were defined based on anatomical structures visualized on corresponding CT images to ensure precise localization. To determine background radioactivity, an ROI was placed in the center of the left ventricular blood pool, avoiding the myocardial wall. Radioactivity concentration within the ROI was quantified as the percentage of injected dose per cubic centimeter (%ID/cc). The tumor‐to‐blood ratio was calculated by dividing the mean %ID/cc of the tumor by the mean %ID/cc of the blood pool.

### 5.7 CAR‐T Cell Lysis and Expansion

The cytolytic activity of CAR‐T cells was evaluated using a luciferase‐based bioluminescence assay. Luciferase‐expressing target cells were seeded in 96‐well plates, followed by the addition of CAR‐T cells at serial effector‐to‐target ratios ranging from 10:1 to 0.3125:1. After 18 h of co‐incubation, Steady‐Glo reagent (Steady‐Glo Luciferase Assay Kit, #E2520, Promega, Madison, WI, USA) was added and incubated for 10 min. Luminescence was measured using an EnVision 2103 Multilabel Reader (PerkinElmer, Shelton, CT, USA), and cytolytic activity was quantified based on the relative reduction in luminescence intensity compared with target‐only controls.

To evaluate the influence of an additional CAR targeting CD19 on cytotoxic potency, CAR‐T cells were preincubated with replication‐incompetent NALM6 cells for 4 h prior to co‐culture with OVCAR3 target cells. Cytolytic activity was determined as described above. To assess the effect of CD19‐targeting on CAR‐T cell expansion, SKOV3 and Raji cells were seeded in 12‐well plates and cultured for 24 h, followed by treatment with 30 mg/mL mitomycin C for 3 h. After washing, CAR‐T cells were added and co‐cultured for 48 or 96 h, and CAR‐T cell numbers were quantified by flow cytometry as described above.

To examine the function of a dnTGFβRII construct on CAR‐T cell cytotoxicity, CAR‐T cells were pretreated for 24 h with either control medium or medium containing 2 ng/mL recombinant human TGFβ1 (#TG1‐H4212, ACRO Biosystems). After removing TGFβ1 via washing, CAR‐T cells were co‐cultured with SKOV3 target cells for 18 h. Thereafter, luciferase substrate (ONE‐Glo Luciferase Assay System, #72056, Promega) was added and incubated for 15 min at room temperature, and luminescence intensity was measured to quantify cytolytic activity.

Additional experimental procedures not described in the main text are detailed in the Supporting Information.

### 5.8 Statistical Analysis and Reproducibility

For bar graphs, data are presented as mean ± SEM, with individual data points shown. Statistical analyses were performed using GraphPad Prism (v.10.6.0) and R (v.4.6.0). The number of mice per group, as well as the number (*n*) and type of replicates (biological or technical), are specified in the corresponding figure legends. Data normality was assessed using the D'Agostino–Pearson, Anderson–Darling, Shapiro–Wilk, or Kolmogorov–Smirnov tests, depending on sample size. *P* values are reported in the figures, and values <0.05 are considered statistically significant.

The following statistical tests were applied: Unpaired Student's *t*‐tests were used to compare means between two independent groups when data were normally distributed; otherwise, Mann–Whitney *U*‐tests were applied. One‐way analysis of variance (ANOVA) was used for comparisons among more than two groups with normally distributed data, followed by Tukey's multiple‐comparison test. When normality was not met, Kruskal–Wallis *H*‐tests were performed with Dunn's post hoc correction. Two‐way repeated‐measures ANOVA was used to analyze tumor growth curves, body weight trajectories, and CAR‐T cell expansion or cytotoxicity over time. This accounted for treatment/genetic modification/TGFβ1 presence and temporal effects, with the Šidák or Tukey's multiple‐comparison correction applied as appropriate. Mixed‐effects models were used for nonparametric longitudinal data or datasets with missing values. Gene Set Enrichment Analysis of transcriptomic data was performed using permutation testing, and multiple testing correction was conducted using the Benjamini–Hochberg method to control the false discovery rate.

Tumor ROIs were defined based on tissue morphology and nuclear staining. For each marker, fixed marker‐specific thresholding parameters were applied uniformly across all groups within the same staining experiment. Marker positivity was calculated as the percentage of threshold‐positive area relative to the total tumor ROI, or the percentage of positive cells among total cells within the analyzed tumor ROI. The quantification reflected ROI‐based marker‐positive percentages. Quantification was performed in a blinded manner by two independent investigators using QuPath (v.0.5.0; https://qupath.github.io/), ImageJ (v.1.54 h), or other software to ensure objectivity.

## Author Contributions

Y. F., Q. G., and G. H. conceived and designed the study. H. L., M. X., J. Liu, X. J., W. M., X. L., Y. X., D. Y., Y. H., S. X., Y. G., and L. Z. contributed to methodology development and experimental design. H. L., M. X., Q. X., J. Liu, X. J., W. M., X. L., K. T., D. Z., and W. G. performed experiments and acquired data. H. L., M. X., and Y. X. conducted formal data analysis and interpretation. Y. F., Q. G., L. Z., G. H., B. Z., and J. Li provided essential resources and technical support. H. L., M. X., and G. H. prepared data visualizations and figures. Y. F., Q. G., L. Z., and G. H. supervised the research and provided strategic direction. Y. F., Q. G., and H. L. acquired funding and oversaw project administration. H. L. and M. X. drafted the original manuscript. All authors contributed to the review and editing of the manuscript and approved the final version for publication.

## Funding

This project was supported by the National Natural Science Foundation of China (82272707, 82441045, and 82072889), the Noncommunicable Chronic Diseases‐National Science and Technology Major Project (2025ZD0545600 and 2025ZD05456001), the National Key Technology Research and Development Program of China (2022YFC2704200 and 2022YFC2704205), the Innovation Group Project of Hubei Province (2023AFA036), the Postdoctoral Fellowship Program and China Postdoctoral Science Foundation under Grant Number BX20250220 and 2025M782358, the Guangdong Basic and Applied Basic Research Foundation (2025A1515110061), and the Chih Kuang Scholarship for Outstanding Young Physician‐Scientists of Sun Yat‐sen University Cancer Center (CKS‐SYSUCC‐2025009).

## Conflicts of Interest

The authors declare no conflicts of interest.

## Supporting information




**Supporting File 1**: advs76994‐sup‐0001‐SuppMat.docx.

**Supporting File 2**: advs76994‐sup‐0002‐TableS3.xlsx.

## Data Availability

The transcriptomic datasets have been deposited in the NCBI Sequence Read Archive (SRA) database under accession number PRJNA1363926. All data generated or analyzed in this study are provided within the article and its Supporting Information. All other raw data supporting the findings of this study are available from the corresponding author upon reasonable request.
