# Supplementary material for: PARP Inhibitors plus Anlotinib as Bridging Therapy for Armored CAR‐T Cells in Ovarian Cancer Enhances Infiltration and Antitumor Efficacy
Source: Adv Sci (Weinh). 2026 Aug 10;13(53):e76994. doi: 10.1002/advs.76994 (PMC13456071; doi:10.1002/advs.76994)
Supplement: Supplementary file 1 [file ADVS-13-e76994-s002.docx]

Supporting Information

PARP Inhibitors plus Anlotinib as Bridging Therapy for Armored CAR-T Cells in Ovarian Cancer Enhances Infiltration and Antitumor Efficacy

Huayi Li, Minghua Xiang, Qiuyang Xu, Jiahao Liu, Xiaofei Jiao, Wei Mu, Xiaoying Lv, Kangjia Tao, Yu Xu, Dongchen Zhou, Wenjian Gong, Danmei Yan, Yixin Hu, Sen Xu, Bingbing Zhao, Jundong Li, Yongkang Gai, Guang Hu, Li Zhu*, Qinglei Gao*, and Yong Fang*

**Text**

*Establishment of OVCAR8-luci cells*: To generate luciferase-expressing OVCAR8 cells (OVCAR8-luci), parental OVCAR8 cells were transduced with a lentiviral vector encoding firefly luciferase (pLV3ltr-eCMV-Luc-PGK-Puro, #P2022062S, Corues Biotechnology, Nanjing, China). After transduction, cells were cultured in complete medium containing 10 μg/mL puromycin for 2 weeks to select stably transduced cells. To preserve stable luciferase expression, OVCAR8-luci cells were periodically maintained under puromycin selection during subsequent passages.

*Mice anesthesia:* Mice were anesthetized with 2% isoflurane in oxygen delivered through a precision vaporizer for procedures requiring immobilization, including retro-orbital blood collection, establishment of cell line-derived xenograft (CDX) and patient-derived xenograft (PDX) models, and in vivo imaging. Anesthesia was administered at the time points specified in corresponding protocols and was maintained for the minimum duration required. For retro-orbital blood collection, anesthesia lasted less than one minute per session. Anesthesia for CDX or PDX implantation was maintained for procedures, lasting less than three minutes. Positron emission tomography-computed tomography and in vivo imaging system (IVIS) required approximately 15 and five minutes of anesthesia, respectively. Mice were monitored for respiratory compromise, delayed recovery, or other signs of distress, and appropriate supportive care was provided as needed in accordance with institutional and national guidelines for animal experimentation.

*IVIS:* Mice received an intraperitoneal injection of D-Luciferin potassium salt at 150 mg/kg (#HY-12591B, MedChemExpress, Monmouth Junction, NJ, USA), prepared in Dulbecco's phosphate-buffered saline (DPBS, #14190144, Thermo Fisher Scientific, Waltham, MA, USA) at a concentration of 15 mg/mL. Bioluminescent signals were acquired 10 minutes after D-Luciferin administration using an IVIS Spectrum imaging system (PerkinElmer, Shelton, CT, USA). Regions of interest were drawn over the abdominal area, and total photon flux (photons/second) was quantified as a measure of tumor burden using Living Image software (v.4.7.3). Imaging parameters, including exposure time, binning, and field of view, were kept consistent across mice and time points within each experiment.

*PDX model:* Fresh tumor specimens were obtained from patients with epithelial ovarian cancer enrolled in a clinical trial (NANT, NCT04507841).^[1]^ The study protocol was reviewed and approved by the Institutional Review Board (S122-5). Tumor tissues were transported to the laboratory in sterile, ice-cold Roswell Park Memorial Institute 1640 medium (#11875093, Gibco, Waltham, MA, USA) supplemented with 1% penicillin-streptomycin (#G4003, ServiceBio, Wuhan, China). Necrotic and fibrotic regions were excised, and viable tumor fragments (approximately 3 × 3 × 3 mm^3^) were subcutaneously implanted into the flanks of 4–6-week-old female NCG mice (one tumor fragment per mouse). Tumor growth was monitored biweekly using digital calipers. When tumors reached around 1,000 mm^3^, mice were euthanized, and xenograft tumors were excised, minced, and serially transplanted into new recipient NCG mice to establish stable PDX lines.

*Safety analysis:* Peripheral blood samples were collected from mice via retro-orbital puncture using sterile capillary tubes. For hematologic evaluation, 100 μL of blood was transferred into EDTA-coated microtubes and analyzed using an automated hematology analyzer (BC-5000vet, Mindray, Shenzhen, China) to determine red and white blood cell counts, platelet counts, and other hematologic indices. For serum preparation, the remaining blood was allowed to clot, followed by centrifugation. The supernatant was analyzed for hepatic and renal function parameters, including alanine aminotransferase and aspartate aminotransferase, using an automatic biochemical analyzer (Chemray 800, Rayto, Shenzhen, China) according to the manufacturer’s instructions.

*Histologic analysis:* Serial 4 μm sections of mouse major organs underwent H&E staining (#G1076, ServiceBio) following the manufacturer’s protocol to assess safety. Tumor sections were processed for Masson’s trichrome staining (#G1006, ServiceBio) and picro-sirius red staining (#G1078, ServiceBio) according to the manufacturer’s instructions to evaluate fibrillar collagen deposition. Images were captured using a high-resolution slide scanning system (#SQS-40P, TEKSQRAY, Shenzhen, China).

*Vascular leakage and perfusion evaluation:* To assess tumor vascular leakiness and perfusion, mice were injected via the tail vein with 100 μL of rhodamine B isothiocyanate-conjugated dextran (25 mg/mL, 70 kDa, #R9379, Sigma-Aldrich, St. Louis, MO, USA) and 100 μL of DyLight 594-conjugated lectin (1 mg/mL, #L32471, Thermo Fisher Scientific). After a 30-minute circulation period to ensure adequate distribution of the tracers, mice were euthanized, and a vertical inline incision along the sternum was performed to expose the heart and lungs. Whole-body perfusion was conducted to remove residual intravascular dye. Specifically, an incision was made in the right atrium, and 10 mL of PBS (#G4202, ServiceBio) containing 2 mmol/L EDTA (#E1170, Solarbio, Beijing, China) was gently perfused through the left ventricle. Tumors were excised, embedded in optimal cutting temperature (OCT) compound (#4583, Sakura Finetek, Tokyo, Japan), and sectioned. The immunofluorescence staining procedures were performed as described below.

*Immunofluorescence:* Paraffin-embedded 4 μm sections were deparaffinized, rehydrated, and subjected to antigen retrieval. Sections were blocked and incubated overnight with primary antibodies diluted to manufacturer-recommended concentrations. Fluorophore-conjugated secondary antibodies were applied to tissues and mounted with coverslips. Nuclei were counterstained with DAPI (#G1012, ServiceBio). Fluorescent images were captured using a digital slide scanner (Pannoramic SCAN, 3DHISTECH, Budapest, Hungary).

*SHG:* Second harmonic generation (SHG) signals arose from noncentrosymmetric structures such as collagen fibrils, in which two incident photons combined to generate a photon of exactly half the excitation wavelength.^[2]^ As tumor-associated collagen fibers act as physical barriers impeding T cell infiltration in solid tumors, we evaluated whether niraparib plus anlotinib could reduce collagen fibrils. OCT-embedded 15μm sections were equilibrated at 37 ℃ for 30 minutes. Nuclear staining was performed with To-Pro-3 (#T3605, Thermo Fisher Scientific) at a 1:1000 dilution for 15 minutes at room temperature, followed by mounting with antifade medium (#ANT061, Antgene, Wuhan, China). Multiphoton SHG imaging was conducted using a ZEISS 710S confocal microscope (Oberkochen, Germany). SHG signals were collected at an excitation wavelength of 820 nm using a 440 ± 30 nm narrow bandpass emission filter.^[3]^

*HTRF:* Homogeneous time-resolved fluorescence (HTRF) assay was conducted to quantify interferon γ (IFNγ) concentrations in co-culture supernatants of chimeric antigen receptor (CAR)-T cells and tumor cells at an effector:target ratio of 5:1. Before co-culture assays, K562-mesothelin (MSLN) and NALM6-MSLN cells were routinely monitored by flow cytometry to confirm stable and homogeneous surface expression of MSLN. Target cells were counted and assessed for viability, and the input cell numbers were adjusted to ensure that equivalent numbers of viable MSLN-positive target cells were used. After 18 hours of co-incubation, cell-free supernatants were collected. IFNγ levels were measured using a commercial HTRF Human IFNγ Detection Kit (62HIFNGPEG, Cisbio Bioassays, Codolet, France) following the manufacturer’s protocol. Briefly, samples and standards were dispensed into 96-well plates and sequentially incubated with anti-IFNγ antibodies labeled with donor and acceptor fluorophores. Following incubation at room temperature to allow antigen-antibody complex formation, time-resolved fluorescence signals were recorded using an EnVision 2103 Multilabel Reader (PerkinElmer).

*Cytokine array:* Cytokine profiling was performed using a customized Mouse Cytokine Antibody Array (RayBiotech, Norcross, GA, USA). The assay was conducted according to the manufacturer's protocol by H-Wayen Biotechnologies (Shanghai, China). In brief, capture antibodies were immobilized at pre-defined positions on the array membrane. Tumor lysates were applied in duplicate, allowing cytokines in the samples to bind their corresponding capture antibodies. Biotinylated detection antibodies were subsequently added to form antibody-antigen-antibody complexes, followed by incubation with a fluorescent dye-conjugated streptavidin for signal development. Fluorescent signals were detected using a GenePix 4000B system (Axon Instruments, Foster City, CA, USA), and densitometric quantification was performed with GenePix Pro 6.0 software (Axon Instruments). For each membrane, cytokine signals were background-subtracted, and the mean signal intensity for each cytokine in each sample was calculated from four technical replicate spots. To correct for inter-membrane technical variation, signal intensities were normalized to the internal positive control spots on each membrane. Statistical comparisons were performed using biological replicate-level normalized signal intensities between each treatment group and the control group. The heatmap was generated using log₂-transformed normalized signal intensities with a common color scale across the entire cytokine panel. Dot plots show normalized signal intensities for selected cytokines, with each dot representing one biological replicate. The raw data of cytokine array are provided in **Table S3**.

*ELISA:* Enzyme-linked immunosorbent assay (ELISA) was conducted to quantify megakaryocytic potentiating factor (MPF) concentrations in human peripheral serum samples. MPF levels were determined using the Human MPF DuoSet ELISA kit (#DY3448, R&D Systems, Minneapolis, MN, USA) following the manufacturer’s instructions. Briefly, standards and serum samples were added in duplicate to pre-coated 96-well plates and sequentially incubated with the capture antibody and horseradish peroxidase-conjugated detection antibody. After color development with tetramethylbenzidine substrate, absorbance was measured at 450 nm using a SpectraMax ABS Plus microplate reader (Molecular Devices, San Jose, CA, USA).

*qPCR:* Genomic DNA (gDNA) was extracted from peripheral blood mononuclear cells using the QIAamp DNA Blood Mini Kit (Qiagen, Hilden, Germany) according to the manufacturer’s protocol. The abundance of CAR-T cells was quantified by quantitative polymerase chain reaction (qPCR) targeting the CAR transgene sequence. A primer-probe set specific to the CAR vector backbone was used to determine vector copy number, with a reference gene serving as an internal control for normalization. Standard curves were generated using serial dilutions of plasmid DNA containing a single copy of the CAR construct to ensure absolute quantification. Reactions were performed using the QuantStudio™ Real-Time PCR System (Applied Biosystems, Foster City, CA, USA) and analyzed with QuantStudio software (Applied Biosystems).

*Transcriptome analysis:* Tumors were preserved in RNA stabilization solution (#AM7201, Thermo Fisher Scientific) and transported on dry ice to Novogene (Beijing, China) for sequencing. Total RNA was extracted using TRIzol reagent (Invitrogen, Carlsbad, CA, USA), and RNA-seq libraries were prepared with the NEBNext® Ultra™ RNA Library Prep Kit for Illumina® (New England Biolabs, Ipswich, MA, USA) according to the manufacturer’s instructions. The library fragments were purified with the AMPure XP system (Beckman Coulter, Brea, CA, USA), and library quality was assessed using the Agilent Bioanalyzer 2100 system (Agilent Technologies, Santa Clara, CA, USA). Libraries were sequenced on NovaSeq platform (Illumina, San Diego, CA, USA) to generate high-quality 150-bp paired end reads. After stringent quality control, reads were aligned to the human reference genome (hg38) using HISAT2, and gene-level counts were quantified with featureCounts (v1.5.0-p3).^[4]^ Differential expression analysis was performed with the DESeq2 package, which estimated log_2_ fold change and adjusted *P* values for each gene.^[5]^ For pathway-level analyses, three predefined gene sets including GOBP_INTERFERON_MEDIATED_SIGNALING_PATHWAY, HALLMARK_HYPOXIA, and GOBP_EXTRACELLULAR_MATRIX_ASSEMBLY were retrieved from the MSigDB database. Gene set enrichment analysis was conducted using the clusterProfiler package,^[6]^ with normalized enrichment scores and adjusted *P* values derived from permutation testing and Benjamini–Hochberg correction. Enrichment outcomes were visualized with clusterProfiler to delineate differential pathway activation between groups.

*Phase 1 clinical trial:* This prospective, single-center, phase 1 clinical trial (NCT05141253) was conducted at Tongji Hospital, Tongji Medical College, HUST to evaluate the safety of the TGFβ-resistant, MSLN/CD19 dual-target CAR-T cells in patients with relapsed or refractory MSLN-positive solid tumors. Key inclusion criteria comprised age ≥ 18 years; receipt of at least two prior lines of standard systemic therapy with no response to the most recent regimen; histologically confirmed MSLN-positive disease; and the presence of at least one measurable lesion according to the Response Evaluation Criteria in Solid Tumors (RECIST, version 1.1).^[7]^ Major exclusion criteria included a history of acute or chronic graft-versus-host disease, prior exposure to gene therapy, and the presence of central nervous system metastases. All participants provided written informed consent prior to enrollment. The study protocol was reviewed and approved by the independent ethics committee of Tongji Hospital and was conducted in accordance with the Declaration of Helsinki, International Council for Harmonization Good Clinical Practice (ICH-GCP) guidelines, and applicable regulatory requirements. The trial followed a standard 3 + 3 dose escalation design. Eligible participants received intravenous CAR-T cells at dose levels of 1 × 10^6^, 3 × 10^6^, or 6 × 10^6^ CAR-T cells/kg following leukapheresis, CAR-T cell manufacturing, and lymphodepletion. Bridging therapy was allowed between apheresis and lymphodepletion at the discretion of the investigators, including but not limited to PARP inhibitors plus anlotinib. The primary endpoints were dose-limiting toxicity, adverse events, and serious adverse events. Secondary endpoints included objective response rate, progression-free survival, pharmacokinetics, and others.

**Figures**

**Figure S1.** PARP inhibitor combined with anlotinib promotes durable T cell infiltration.

**A**. Representative gross images of ID8 tumors excised from the indicated treatment groups at day 21. Scale bar, 1 cm. Ctrl denotes vehicle; Nira denotes niraparib; Anlo denotes anlotinib; and Comb denotes the combination of niraparib and anlotinib. **B**. Flow cytometry gating strategy for tumor-infiltrating T cell analysis. Following sequential gating of singlets, live cells, and CD45⁺ CD3⁺ T cells, CD4 and CD8 expression was used to identify CD4⁺ CD8⁻ single-positive (the lower-right quadrant), CD4⁺ CD8⁺ double-positive (the upper-right quadrant), CD4⁻ CD8⁺ single-positive (the upper-left quadrant), and CD4⁻ CD8⁻ double-negative (the lower-left quadrant) T cell populations. Within CD45⁺ CD3⁺ T cells, quantitative analyses focused on the CD4⁺ CD8⁻ and CD4⁻ CD8⁺ single-positive subsets. **C** and **D**. Flow cytometric quantification of intratumoral CD4^+^ (**C**) and CD8^+^ (**D**) T cells as a percentage of live cells at day 14 after withdrawal. *n* = 7 biologically independent mice per group. Statistical significance was assessed by one-way analysis of variance (ANOVA) with Tukey’s post hoc multiple-comparison test. **E**. Longitudinal monitoring of body weight. *n* = 10–12 per group. Data were analyzed using two-way repeated-measures ANOVA (Comb vs. Ctrl or Anlo) or a mixed-effects model with restricted maximum likelihood (REML) estimation (Comb vs. Nira). **F**. Safety evaluation of peripheral blood markers including alanine transaminase (ALT), aspartate transaminase (AST), blood urea nitrogen (BUN), creatinine, white blood cell (WBC), neutrophils, lymphocytes, red blood cells (RBC), platelets, and hemoglobin. *n* = 6 per group. Statistical significance was determined by one-way ANOVA. **G**. Representative H&E staining of the heart, kidney, liver, lung, and spleen of mice at day 21. **H**. Schematic of the experimental design in ID8 tumor-bearing mice treated with olaparib. **I** and **J**. Representative immunohistochemical images (**I**) and quantification (**J**) of intratumoral CD3^+^ T cell (brown) abundance among total cells at day 0 (left), day 7 (middle), and day 14 (right) following withdrawal. Nuclei were counterstained with hematoxylin (blue). *n* = 5 per group. Statistical significance was determined using a two-tailed unpaired Student’s *t*-test. Ctrl denotes vehicle and Ola denotes olaparib. Data are presented as mean ± SEM (**C**–**F** and **J**). Scale bars, 100 μm (**G** and **I**).

**Figure S2.** Niraparib and anlotinib synergistically activate the cGAS-STING signaling axis.

**A**–**F**. Immunofluorescence staining (left) and quantification (right) of intratumoral γH2AX (**A** and **B**), pTBK1 (**C** and **D**), and CXCL10 (**E** and **F**) (green) signals at day 0 and day 7 following treatment withdrawal. *n* = 4–6 biologically independent mice per group. Ctrl denotes vehicle; Nira denotes niraparib; Anlo denotes anlotinib; and Comb denotes the combination of niraparib and anlotinib. **G**–**I**. Immunofluorescence staining (left) and quantification (right) of CCL5 (green) abundance in tumor sections at day 0 (**G**), day 7 (**H**), and day 14 (**I**) following withdrawal. *n* = 5–7 per group. For **A**–**I**, statistical significance was determined using one-way analysis of variance (ANOVA) followed by Tukey’s post hoc multiple-comparison test. Nuclei were counterstained with DAPI (blue), and boxed areas were further magnified. Data are presented as mean ± SEM (**A**–**I**). Scale bars, 100 μm (**A**–**I**).

**Figure S3.** Niraparib plus anlotinib promotes functional normalization of tumor vasculature.

**A**–**C**. Representative immunofluorescence staining (left) of CD31 (red) and corresponding quantification (right) of vascular density in tumor sections at day 0 (**A**), day 7 (**B**), and day 14 (**C**) following treatment withdrawal. *n* = 6 or 7 biologically independent mice per group. Ctrl denotes vehicle; Nira denotes niraparib; Anlo denotes anlotinib; and Comb denotes the combination of niraparib and anlotinib. **D**–**O**. Immunofluorescence co-staining (left) of CD31 (red) with PDGFRβ (**D** and **E**), claudin-5 (**F** and **G**), Col IV (**H** and **I**), dextran (**J** and **K**), lectin (**L** and **M**), or CA IX (**N** and **O**) (green) and quantification (right) of pericyte coverage (**D** and **E**), claudin-5^+^ vessel (**F** and **G**), basement membrane (BM) integrity (**H** and **I**), vascular leakage (dextran, **J** and **K**), vascular perfusion (lectin, **L** and **M**), or hypoxic regions (**N** and **O**) in tumor sections at day 0 and day 7 following withdrawal. *n* = 5–7 per group. For panels **A**–**O**, statistical significance was determined using one-way analysis of variance (ANOVA) followed by Tukey’s post hoc multiple-comparison test. Nuclei were counterstained with DAPI (blue). Data are presented as mean ± SEM (**A**–**O**). Scale bars, 100 μm (**A**–**O**).

**Figure S4.** Bridging therapy alleviates aberrant fibrillar collagen fiber deposition within the TME.

**A** and **B**. Representative SHG imaging (left) and quantification (right) of intratumoral fibrillar collagen fiber density at day 0 (**A**) and day 7 (**B**) following withdrawal. Fibrillar collagen fibers are shown in white, and nuclei were stained with To-Pro-3 (red). *n* = 5 biologically independent mice per group. Statistical significance was assessed using one-way analysis of variance (ANOVA) with Tukey’s post hoc multiple-comparison test. Ctrl denotes vehicle; Nira denotes niraparib; Anlo denotes anlotinib; and Comb denotes the combination of niraparib and anlotinib. SHG, second harmonic generation. **C**–**H**. Representative images (left) and quantification (right) of fibrillar collagen fiber density assessed by Masson’s trichrome staining (collagen fibers were stained blue, **C**–**E**) or picro-sirius red staining (collagen fibers were stained red, **F**–**H**) at day 0 (**C** and **F**), day 7 (**D** and **G**), and day 14 (**E** and **H**) following withdrawal. *n* = 7 per group. Statistical significance was determined using one-way ANOVA with Tukey’s post hoc multiple-comparison test (**C**, **E**, and **H**) or Kruskal–Wallis *H*-test with Dunn’s post hoc multiple-comparison test (**D**, **F**, and **G**). Data are presented as mean ± SEM (**A**–**H**). Scale bars, 100 μm (**A**–**H**).

**Figure S5.** Post-treatment cytokine landscape reveals an immunoreactive TME.

**A**. Heatmap of log_2_-transformed mean normalized cytokine signal intensities, depicting intratumoral cytokine abundance across groups. *n* = 4 biologically independent mice per group. Statistical significance was assessed by a two-tailed unpaired Mann–Whitney *U*-test comparing each treatment group with the control group. Ctrl denotes vehicle; Nira denotes niraparib; Anlo denotes anlotinib; and Comb denotes the combination of niraparib and anlotinib. **P* < 0.05. **B**. Representative cytokine array membrane images showing cytokine distribution. PC, positive control. **C**. Dot plots showing normalized signal intensities of selected cytokines. Statistical significance was assessed by two-tailed unpaired Mann–Whitney *U*-test. *n* = 4 per group. Data are presented as mean ± SEM.

**Figure S6.** Cell preparations.

**A**. Schematic of CAR-T cell architecture. **B**–**E**. Representative flow cytometry dot plots showing the percentages of MSLN-positive cells among live empty vector-transduced and MSLN-expressing K562 (**B**) and NALM6 (**D**) cells. Representative fluorescence-intensity histograms showing surface MSLN expression in MSLN-expressing cells, the corresponding empty vector controls, and unstained controls for K562 (**C**) and NALM6 (**E**) cells. Median fluorescence intensity (MFI) values are shown as ×10^3^.

**Figure S7.** TGFβ-resistant MSLN/CD19 dual-target CAR-T cells exhibit enhanced antitumor efficacy in vivo.

**A**. Longitudinal monitoring of body weight in mice bearing subcutaneous SKOV3 tumor treated with a high dose of CAR-T cells. *n* = 6 biologically independent mice per group. Statistical analyses were performed using a mixed-effects model with restricted maximum likelihood (REML) estimation; post hoc comparisons at individual timepoints were conducted with Tukey’s multiple-comparison test. ns, not significant. UTD, untransduced T cells; PBS, phosphate-buffered saline. **B**. Kinetics of circulating CAR-T cells in peripheral blood. *n* = 6 per group. **C**. Longitudinal body weight monitoring in mice bearing subcutaneous SKOV3 tumor treated with a low dose of CAR-T cells. *n* = 6 per group. Data were analyzed using a mixed-effects model with REML estimation. **D**. Kinetics of circulating CAR-T cells in peripheral blood. *n* = 6 per group. Data are presented as mean ± SEM (**A** and **C**) or as median (**B** and **D**).

**Figure S8.** Bridging therapy enhances CAR-T cell antitumor activity in subcutaneous and intraperitoneal OVCAR8 tumor models.

**A**. Schematic of the experimental design for subcutaneous (s.c.) OVCAR8 tumor model. **B**. Tumor growth curves. *n* = 7 biologically independent mice per group. Data were analyzed using a mixed-effects model with restricted maximum likelihood (REML) estimation, followed by Tukey’s multiple-comparison test at the indicated time points. The *P* value indicated at day 21 corresponds to the comparison between the Vehicle + CAR-T and Treat + CAR-T groups. Vehicle + UTD denotes vehicle followed by untransduced T cells (UTD); Treat + UTD denotes bridging therapy followed by UTD; Vehicle + CAR-T denotes vehicle followed by CAR-T cells; and Treat + CAR-T denotes bridging therapy followed by CAR-T cells. **C**. Representative images of tumors excised from mice in the four groups at experimental endpoint. Scale bar, 1 cm. **D**. Longitudinal monitoring of body weight in mice bearing s.c. OVCAR8 tumors. *n* = 7 per group. Data were analyzed by a mixed-effects model with REML estimation. **E**. Schematic of the experimental design for intraperitoneal (i.p.) OVCAR8-luci tumor model. **F** and **G**. Longitudinal IVIS bioluminescence imaging of mice bearing i.p. OVCAR8-luci tumors (**F**) and quantification (**G**) of tumor-associated bioluminescence signals at the indicated time points. *n* = 5 per group. Data were analyzed by two-way repeated-measures analysis of variance (ANOVA) followed by Šidák multiple-comparison test. Ctrl denotes vehicle followed by CAR-T cells, whereas BT denotes bridging therapy followed by CAR-T cells. **H**. Representative gross images of i.p. OVCAR8-luci tumors at the experimental endpoint. Outlined regions indicate tumor deposits on the diaphragm. **I**. Longitudinal monitoring of body weight in mice bearing i.p. OVCAR8-luci tumors. *n* = 5 per group. Data were analyzed by two-way repeated-measures ANOVA. Data are presented as mean ± SEM (**B**, **D**, **G**, and **I**).

**Figure S9.** Bridging therapy potentiates CAR-T cell infiltration and antitumor activity.

**A**. Individual tumor growth curves for mice in Ctrl (left) and BT groups (right). *n* = 7 biologically independent mice (Ctrl), *n* = 8 (BT). Ctrl denotes vehicle followed by CAR-T cells, whereas BT denotes bridging therapy followed by CAR-T cells. **B**. Tumor weight at endpoint. *n* = 7 (Ctrl), *n* = 8 (BT). Statistical significance was determined using a two-tailed unpaired Student’s *t*-test. **C**. Representative immunohistochemical images (left) and quantification (right) of intratumoral GZMB^+^ areas (brown). Nuclei were counterstained with hematoxylin (blue), and boxed areas were further magnified. *n* = 7 (Ctrl), *n* = 8 (BT). Data were analyzed by two-tailed unpaired Student’s *t*-test. **D**. Flow cytometry gating strategy for tumor-infiltrating CAR-T cell analysis. **E**. Longitudinal body weight monitoring. *n* = 7 (Ctrl), *n* = 8 (BT). Data were analyzed by two-way repeated-measures analysis of variance. **F**–**H**. Safety evaluation of peripheral blood markers including alanine transaminase (ALT, **F**, left), aspartate transaminase (AST, **F**, right), blood urea nitrogen (BUN, **G**, column 1), creatinine (**G**, column 2), lactate dehydrogenase (LDH, **G**, column 3), creatine kinase (CK, **G**, column 4), white blood cell (WBC, **H**, left), neutrophils (**H**, middle), and platelets (**H**, right). *n* = 6 per group. Statistical significance was determined using two-tailed unpaired Student’s *t*-test or Mann–Whitney *U*-test. **I**. Individual tumor growth curves for mice in Ctrl (left) and BT groups (right). *n* = 7 (Ctrl), *n* = 10 (BT). **J**. Tumor weight at endpoint. *n* = 7 (Ctrl), *n* = 9 (BT). Data were analyzed using a two-tailed unpaired Mann–Whitney *U*-test. **K**. Representative immunohistochemical images (left) and quantification (right) of intratumoral GZMB^+^ areas (brown). Nuclei were counterstained with hematoxylin (blue), and boxed areas were further magnified. *n* = 7 (Ctrl), *n* = 9 (BT). Statistical analysis was performed using a two-tailed unpaired Student’s *t*-test. **L**. Longitudinal body weight monitoring. *n* = 7 (Ctrl), *n* = 10 (BT). Data were analyzed using a mixed-effects model with restricted maximum likelihood (REML) estimation; post hoc comparisons at individual timepoints were assessed with Šidák multiple-comparison test. **M**. GSEA of transcriptomic signatures associated with interferon-mediated signaling (left), hypoxia (middle), and extracellular matrix assembly (right) in tumors following bridging therapy. *n* = 4 per group. Statistical significance was determined by permutation testing; results were presented as NES with adjusted *P* values corrected by the Benjamini–Hochberg method. NES, normalized enrichment score. Data are presented as mean ± SEM (**B**, **C**, **E**–**H**, and **J**–**L**). Scale bars, 100 μm (**C** and **K**).

**Figure S10.** Translational relevance of bridging therapy in a clinically representative ovarian cancer model.

**A**. Individual tumor growth curves for mice in Ctrl (left) and BT groups (right). *n* = 7 biologically independent mice (Ctrl), *n* = 8 (BT). Ctrl denotes vehicle followed by CAR-T cells, whereas BT denotes bridging therapy followed by CAR-T cells. **B** and **C**. Representative immunohistochemical images (**B**) and quantification (**C**) of GZMB^+^ (left, at endpoint) or MSLN^+^ (right, at the time of CAR-T cell infusion) areas (brown). Nuclei were counterstained with hematoxylin (blue). *n* = 6–8 per group. Statistical analysis was performed using two-tailed unpaired Student’s *t*-test. **D**. Longitudinal body weight measurements. *n* = 7 (Ctrl), *n* = 8 (BT). Data were analyzed by two-way repeated-measures analysis of variance (ANOVA) with Šidák post hoc multiple comparison at individual timepoints. **E**. Flow cytometry gating strategy for analysis of tumor-infiltrating CAR-T cells. **F**. Flow cytometry plots (left) and quantification (right) of CAR-T cells as a percentage of live splenocytes. *n* = 6 per group. Statistical analysis was performed using two-tailed unpaired Mann–Whitney *U*-test. **G** and **H**. Immunofluorescence staining (**G**) of CCL5 (green, left) and CD31 (red, right), with quantification (**H**) of CCL5^+^ areas (left) and blood vascular density (right). Nuclei were counterstained with DAPI (blue). *n* = 6 per group. Statistical significance was determined using two-tailed unpaired Student’s *t*-test. **I** and **J**. Representative images (**I**) and quantification (**J**) of tumor fibrillar collagen fiber density assessed by second harmonic generation (SHG) imaging (left), Masson’s trichrome staining (middle), and picro-sirius red staining (right). Fibrillar collagen fibers are shown in white (left), blue (middle), or red (right), and nuclei were stained with To-Pro-3 (red, left). *n* = 5–8 per group. Statistical significance was determined using two-tailed unpaired Student’s *t*-test or Mann–Whitney *U*-test. Data are presented as mean ± SEM (**C**, **D**, **F**, **H**, and **J**). Scale bars, 100 μm (**B**, **G**, and **I**).

**Figure S11.** CAR-T cells expand and are associated with antigen clearance following bridging therapy in a phase I trial.

**A**. Serum kinetics of MPF concentrations in subject 01. Data are presented as mean ± SEM. **B** and **C**. Peripheral expansion kinetics of CAR-T cells in subject 01, assessed by flow cytometry (**B**) and qPCR (**C**). VCN, vector copy number. **D**. Serum kinetics of B cells in subject 01 assessed by flow cytometry.

**Tables**

**Table S1.** Antibodies.

| Antibodies | Source | Identifier |
| --- | --- | --- |
| Anti-mouse CD3ε | Cell Signaling Technology | Cat# 99940; RRID: AB_2755035 |
| Anti-mouse CD4 | Abcam | Cat# ab183685; RRID: AB_2686917 |
| Anti-mouse CD8α | Abcam | Cat# ab217344; RRID: AB_2890649 |
| Anti-human CD3ε | Cell Signaling Technology | Cat# 85061; RRID: AB_2721019 |
| Anti-human CD4 | Abcam | Cat# ab133616; RRID: AB_2750883 |
| Anti-human CD8α | Cell Signaling Technology | Cat# 85336; RRID: AB_2800052 |
| Anti-human granzyme B | Abcam | Cat# ab255598; RRID: AB_2860567 |
| HRP-labeled goat anti-rabbit IgG (H+L) | ServiceBio | Cat# GB23303; RRID: AB_2811189 |
| Anti-Histone H2A.X, phospho (Ser139) | Abcam | Cat# ab81299; RRID: AB_1640564 |
| Anti-phospho-TBK1/NAK (Ser172) | Cell Signaling Technology | Cat# 5483; RRID: AB_10693472 |
| Anti-CCL5 (RANTES) | Thermo Fisher Scientific | Cat# 710001; RRID: AB_2532515 |
| Anti-CXCL10 | Thermo Fisher Scientific | Cat# 701225; RRID: AB_2532429 |
| Anti-mouse CD31 | Cell Signaling Technology | Cat# 77699; RRID: AB_2722705 |
| Anti-PDGFRβ | Thermo Fisher Scientific | Cat# MA5-15143; RRID: AB_10985851 |
| Anti-mouse Collagen IV | GeneTex | Cat# GTX19808; RRID: AB_423741 |
| Anti-mouse Claudin-5 | IBL - America | Cat# 18855; RRID: AB_2276476 |
| Anti-CA9 | Abcam | Cat# ab270401; RRID: AB_3665691 |
| APC/Fire™ 750 anti-mouse CD45 | BioLegend | Cat# 103154; RRID: AB_2572116 |
| APC anti-mouse CD3ε | BioLegend | Cat# 100312; RRID: AB_312677 |
| PerCP anti-mouse CD4 | BioLegend | Cat# 100432; RRID: AB_893323 |
| BD Horizon™ BB515 Rat anti-mouse CD8α | BD Biosciences | Cat# 564422; RRID: AB_2738801 |
| APC/Cyanine7 anti-human CD45 | BioLegend | Cat# 304014; RRID: AB_314402 |
| PE anti-human CD3 | BioLegend | Cat# 300308; RRID: AB_314044 |
| PE/Cyanine7 anti-human CD4 | BioLegend | Cat# 300512; RRID: AB_314080 |
| Brilliant Violet 421™ anti-human CD8 | BioLegend | Cat# 344748; RRID: AB_2629584 |
| Anti-human mesothelin | Abcam | Cat# ab196235; RRID: AB_3665950 |
| Alexa Fluor® 488 goat anti-rabbit IgG H&L | Abcam | Cat# ab150077; RRID: AB_2630356 |
| TruStain FcX™ anti-mouse CD16/32 | BioLegend | Cat# 101320; RRID: AB_1574975 |
| Human TruStain FcX™ | BioLegend | Cat# 422302; RRID: AB_2818986 |

**Table S2.** Flow cytometry expression profiles of engineered CAR-T cell products.

| Cell product | MSLN-CAR positive | CD19-CAR positive | MSLN-CAR/CD19-CAR double-positive | dnTGFβRII positive |
| --- | --- | --- | --- | --- |
| Untransduced T cells | Negative | Negative | Negative | Negative |
| MSLN-CAR-T | ~70% | Negative | Negative | Negative |
| dnTGFβRII-MSLN-CAR-T | ~70% | Negative | Negative | 27.6% |
| CD19-CAR-T | Negative | ~21.6% | Negative | Negative |
| CD19-MSLN-CAR-T | ~60% | ~12.24% | 27.5% | Negative |
| dnTGFβRII-CD19-MSLN-CAR-T | ~58% | ~9.9% | 26.7% | 20.7% |

**Table S3.** Raw data of cytokine array.

Table S3 is uploaded as an Excel file.

**References**

1.	Y. Luo, Y. Xia, D. Liu, et al., “Neoadjuvant PARPi or chemotherapy in ovarian cancer informs targeting effector Treg cells for homologous-recombination-deficient tumors,” *Cell* 187 (2024): 4905–4925.e4924, https://doi.org/10.1016/j.cell.2024.06.013.

2.	X. Chen, O. Nadiarynkh, S. Plotnikov, P. J. Campagnola, “Second harmonic generation microscopy for quantitative analysis of collagen fibrillar structure,” *Nature Protocols* 7 (2012): 654–669, https://doi.org/10.1038/nprot.2012.009.

3.	X. Sun, B. Wu, H. C. Chiang, et al., “Tumour DDR1 promotes collagen fibre alignment to instigate immune exclusion,” *Nature* 599 (2021): 673–678, https://doi.org/10.1038/s41586-021-04057-2.

4.	Y. Liao, G. K. Smyth, W. Shi, “featureCounts: an efficient general purpose program for assigning sequence reads to genomic features,” *Bioinformatics* 30 (2014): 923–930, https://doi.org/10.1093/bioinformatics/btt656.

5.	M. I. Love, W. Huber, S. Anders, “Moderated estimation of fold change and dispersion for RNA-seq data with DESeq2,” *Genome Biology* 15 (2014): 550, https://doi.org/10.1186/s13059-014-0550-8.

6.	G. Yu, L. G. Wang, Y. Han, Q. Y. He, “clusterProfiler: an R package for comparing biological themes among gene clusters,” *OMICS: A Journal of Integrative Biology* 16 (2012): 284–287, https://doi.org/10.1089/omi.2011.0118.

7.	E. A. Eisenhauer, P. Therasse, J. Bogaerts, et al., “New response evaluation criteria in solid tumours: revised RECIST guideline (version 1.1),” *European Journal of Cancer* 45 (2009): 228–247, https://doi.org/10.1016/j.ejca.2008.10.026.
